# Bio-orthogonal click-targeting nanocomposites for chemo-photothermal synergistic therapy in breast cancer

**DOI:** 10.7150/thno.42445

**Published:** 2020-04-06

**Authors:** Jianan Qiao, Fengchun Tian, Yudi Deng, Yunkai Shang, Shijie Chen, Enhao Chang, Jing Yao

**Affiliations:** State Key Laboratory of Natural Medicines and Jiangsu Key Laboratory of Drug Stability of Biopharmaceuticals, Department of Pharmaceutics, China Pharmaceutical University, 24 Tongjiaxiang, Nanjing 210009, China.

**Keywords:** nanocomposite, chemo-photothermal synergistic therapy, metabolic glycoengineering, bio-orthogonal click chemistry, breast cancer

## Abstract

Chemo-photothermal synergistic treatment has a high potential to complement traditional cancer therapy and amplify its outcome. Precision in the delivery of these therapeutic agents to tumor cells has been indicated as being key to maximizing their therapeutic effects.

**Method**: We developed a bio-orthogonal copper-free click-targeting nanocomposite system (DLQ/DZ) that markedly improved specific co-delivery of the chemotherapeutic agent doxorubicin and the photosensitizer zinc phthalocyanine to breast cancer cells *via* a two-step mechanism. In the first step, an azide-modified sugar (tetraacetylated N-azidoacetyl-D-mannosamine, Ac4ManNAz) was injected intratumorally for glycoengineering of the tumor cell surface. Subsequently, DLQ/DZ was administered to achieve tumor enrichment *via* bio-orthogonal copper-free click-targeting.

**Results**: During the first step in our experiments, high density azide groups (3.23×10^7^/cell) were successfully glycoengineered on the surface of tumor cells following Ac4ManNAz administration* in vitro*. Subsequently, the highly efficient bio-orthogonal click chemical reaction between receptor-like azide groups on tumor cells and DBCO on nanocomposites significantly enhanced the cellular uptake and tumor-specific distribution (4.6x increase) of the nanocomposites *in vivo*. Importantly, Ac4ManNAz+DLQ/DZ treatment augmented the anti-cancer effect of combined chemotherapy and PTT (96.1% inhibition rate), nearly ablating the tumor.

**Conclusions**: This bio-orthogonal click-targeting combination strategy may provide a promising treatment approach for surficial breast cancers.

## Introduction

Over the past few decades, the benefits of phototherapy have been demonstrated as an anti-cancer treatment that is non-invasive, has high spatiotemporal precision (limited mainly to the irradiated site), and low side effects 1-5. In particular, photothermal therapy (PTT) utilizes the near-infrared (NIR, 650-900 nm) laser irradiation on the tumor site to kill tumor cells by producing local heat with the assistance of photosensitizers 6-10. This strategy often contributes to very significant therapeutic effects in the early stage. However, the residual tumor cells surviving from uneven temperature distribution in the tumor tissues may lead to recurrence 11-13. It has been reported that solid tumor chemotherapy in combination with PTT effectively kills the remaining tumor cells caused by uneven temperature distribution or insufficient laser exposure during PTT 4, 14-17. More importantly, PTT enables a superior delivery of drugs deep into the tumor *via* expanded endothelial gaps, improved vascular perfusion 18-21, and increases the tumor drug accumulation by enhanced vascular permeabilization 22. PTT also enhances tumor sensitivity to chemotherapeutics 23-26. As such, the combination of chemotherapy and PTT has been shown to complement and amplify therapeutic effects. Precise co-delivery of chemotherapeutic agents and photosensitizers to the tumor whilst avoiding off-target exposure is the key to achieving optimal efficacy 25. Nevertheless, conventional targeting strategies 27 that rely on the interaction between nanocarriers and tumor-specific receptors often result in sub-optimal outcomes. The problem is linked to the limited and varied numbers of endogenous (e.g. protein-based) receptors displayed on tumor cells 28, 29.

Metabolic glycoengineering dresses tumor cells with artificial receptors. The engineering utilizes the intrinsic metabolism of cancer cells and forces cells to incorporate the chemical groups of non-native sugars, such as azide-containing mannosamine derivatives, into their surfaces 30-32. The amount of unnatural sugars administered to the tumor cells can be adjusted to control the density of chemical groups on the cell surface 33, 34. Moreover, the expression of chemical groups as artificial receptors could be manually controlled and exclusively presented on tumor cells 35, which helps to reduce toxic side effects. Subsequently, cyclooctyne-modified nanocarriers may bind to the tumor surface *via* highly specific bio-orthogonal copper-free click chemical reactions, thereby, efficiently and accurately delivering antitumor agents 36.

It was reported that the number of “receptor-like” azide groups produced on the tumor cell surface by metabolic glycoengineering was 100-times higher than the estimated number of endogenous receptors 37. Furthermore, cellular uptake of cyclooctyne-modified nanoparticles 33 based on metabolic glycoengineering and click reaction in human lung cancer (A549) cells was increased in about 10-fold compared to the nanoparticles delivered *via* endogenous receptor-mediated targeting 38, 39. Therefore, the combination of metabolic glycoengineering and bio-orthogonal click chemistry may provide a better instrument for tumor-targeted drug delivery.

**Scheme 1:**
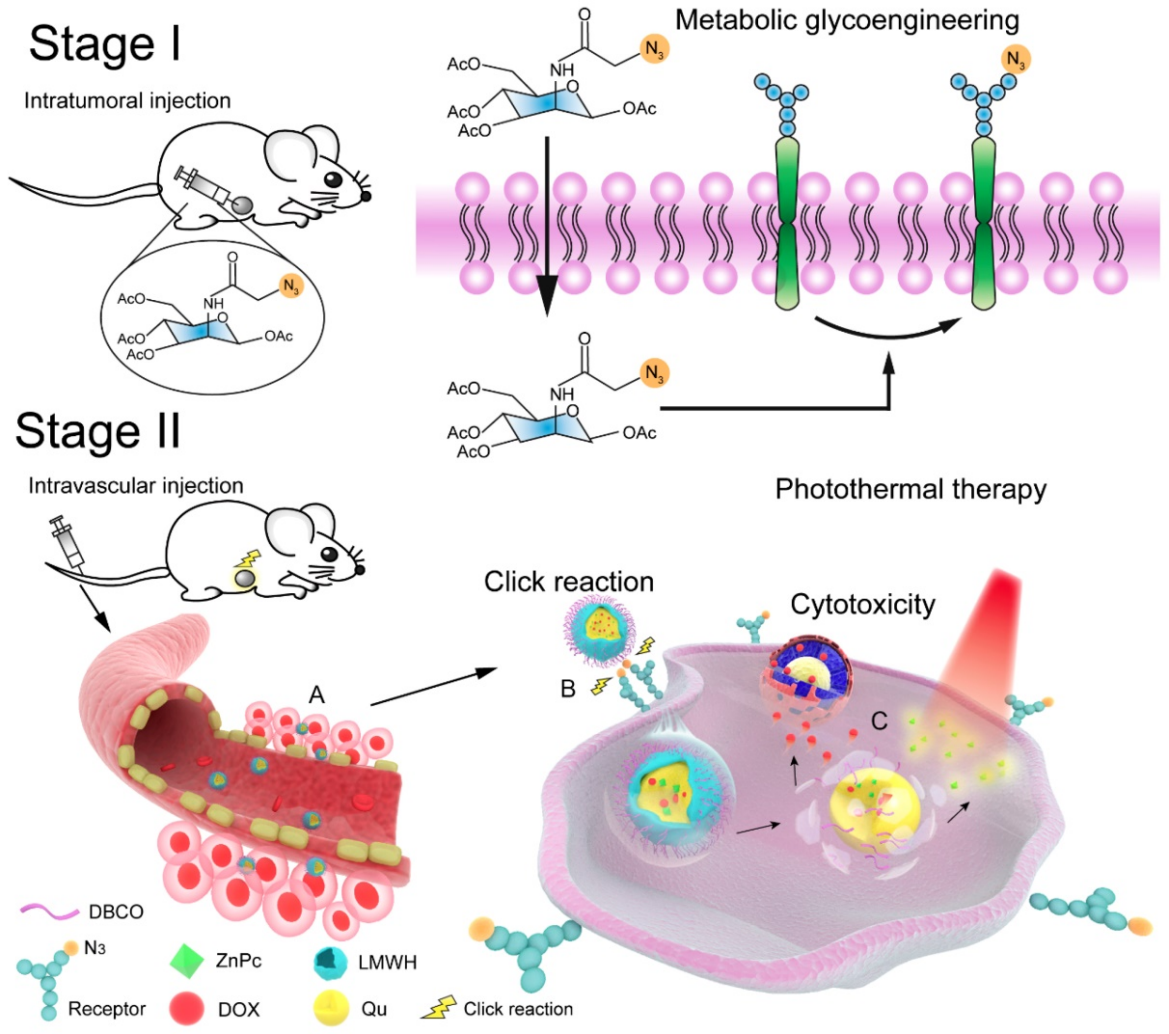
Schematic engineered of the click-targeting nanocomposites for chemo-photothermal synergistic therapy. Stage I: Production of azide groups on the surface of tumor cells by metabolism glycoengineering. Stage II: (A) Nanocomposites accumulated in tumor tissue through EPR effect. (B) Nanocomposites bound to tumor cell *via* bio-orthogonal copper-free click chemistry. (C) Chemotherapy combined with photothermal therapy to synergize anti-tumor by DNA damage of doxorubicin (DOX) and thermal ablation of zinc phthalocyanine (ZnPc) upon laser irradiation.

In the current study, a bio-orthogonal click-targeting nanocomposite was constructed to enable the synchronous delivery of photothermal conversion agent (zinc phthalocyanine, ZnPc) and chemotherapeutic drug (doxorubicin, DOX) to lesion sites and to realize synergistic combination therapy of PTT and chemotherapy. We have previously demonstrated that low molecular weight heparin (LMWH)-quercetin (Qu) conjugate (LQ) is an efficient carrier for drug delivery and inhibits the activity and expression of P-glycoprotein on tumor cells 40. In this case, dibenzocyclooctyne (DBCO) modified LQ conjugates (DLQ) were synthesized and self-assembled into nanocomposites in aqueous medium to co-encapsulate DOX and ZnPc through hydrophobic and π-π stacking interactions (i.e. DLQ/DZ). Tetraacetylated N-azidoacetyl-D-mannosamine (Ac4ManNAz) was used to engineer azide-rich tumor cell surface. As illustrated in Scheme 1, *i.t.* injection of Ac4ManNAz into the tumor site glycoengineered a large number of receptor-like azide groups distributed on the tumor cell surface. Subsequently, DLQ/DZ which accumulated in tumor *via* tumor-specific enhanced permeability and retention (EPR) effect could bind to highly-expressed azide groups on surface of tumor cells through the bio-orthogonal copper-free click chemistry reactions after *i.v.* injection, facilitating their internalization into tumor cells effectively. Following this, the esterase or acidic tumor environment hydrolyzed the ester bond of DLQ to release both ZnPc and DOX. ZnPc was irradiated by NIR laser to induce the rise in temperature inside tumor cell 41 and trigger tumor cell protein denaturation and coagulative necrosis to cause irreversible apoptosis. Activated cell death mechanisms reduced tumor resistance to chemotherapeutic drugs. Synergizing with ZnPc, DOX was concentrated in cell nuclei, blocked DNA synthesis, and inhibited tumor cell proliferation 42, 43. Thus, the targeted nanocomposite utilized the autologous metabolic mechanism of tumors to achieve the efficient delivery of photosensitizer and chemotherapeutic drug. The combined physical and chemical treatments markedly enhanced the antitumor efficacy of used drugs, meanwhile maintaining a promising safety profile in the animal model. In this study, tumor-targeting drug delivery and chemo-photothermal therapeutic efficacy of DLQ/DZ nanocomposites were evaluated *in vitro* and *in vivo*.

## Materials and Methods

### Materials

Low molecular weight heparin (100 IU mg^-1^, MW 4500 Da) was provided by Nanjing University (Nanjing, China). Quercetin was purchased from Sanwei Pharmaceutical Co., Ltd (Shanghai, China). Dox was purchased from Kaifang Pharmaceutical Technology Co., Ltd (Shanghai, China). ZnPc was obtained from Sigma-Aldrich (Shanghai, China). N-methylpyrrolidone (NMP), 1-Ethyl-3 (3-dimethylaminopropyl) carbodiimide (EDC) and N-hydroxysuccinimide (NHS) were obtained from Aladdin Biochemical Technology Co., Ltd (Shanghai, China). DBCO-Cy5 and Ac4ManNAz were purchased from Click Chemistry Tools (Scottsdale, USA). Cy5-NH_2_ was purchased from Xi'an ruixi Biological Technology Co., Ltd (Shanxi, China). Other chemicals were of analytical grade and were used without further purification.

### Synthesis and characterization of DLQ conjugates

DLQ conjugates were synthesized connecting Qu and DBCO into LMWH backbone *via* ester and amide bonds, respectively. DBCO-NH_2_ synthesis method was reported in Supplementary Information section (Figure S1-7). Firstly, LMWH (1.0 mmol) was mixed to react with NHS (4.0 mmol) and EDC (4.0 mmol) in anhydrous formamide at 4 ^o^C for 1 h. Afterwards, DBCO-NH_2_ (2.0 mmol) solution was added and mixture was stirred at 25^ o^C for 24 h. The reaction solution was quickly poured into subcooled acetone (5-10 volumes) and precipitated at -20 ^o^C for 30 min. The precipitate was extracted by suction-forced filtration, dissolved in distilled water, and the resulting solution was dialyzed in MWCO=3500 dialysis bag for 2 days. DBCO-LMWH (DL) conjugates were obtained by lyophilization. The Qu was grafted onto the DL to obtain DLQ conjugates by the same method as the preparation of DL conjugates. The molar grafting ratio of DBCO (308 nm) and Qu (360 nm) into LMWH were calculated using UV-Vis spectroscopy according to the following formula.

The molar grafting ratio (%) = (c / M_X_) / [(m - c) / M_LMWH_] × 100%

c was the content of Qu or DBCO, M_X_ was the molecular weight of Qu or DBCO, m was the mass of the conjugate, and M_LMWH_ was the molecular weight of a low molecular weight heparin structural unit.

Various amounts of N-(2-aminoethyl)-2-azidoacetamide hydrochloride (10%, 20%, 30%, 50%, 70%) were added to the DLQ conjugate solution at room temperature for 2 h, and then subjected to lyophilization. The lyophilized products were ground with KBr, and then compressed. The infrared spectrum was recorded by infrared spectrometer (FT-IR nicolet impact 410). The degree of reaction between the azide group of N-(2-aminoethyl)-2-azidoacetamide hydrochloride and DBCO of DLQ was observed within FT-IR spectrum.

### Synthesis of DLQ-Cy5

Cy5 was connected to DLQ *via* an amide bond. Firstly, DLQ (1.0 mmol) had been reacting with NHS (4.0 mmol) and EDC (4.0 mmol) in anhydrous formamide at 4 ^o^C for 1 h. Following this, Cy5-NH_2_ (2.0 mmol) solution was added and stirred at 25^ o^C for 24 h. Thereafter, the reaction solution was quickly poured into subcooled acetone (5-10 volumes) and precipitated at -20 ^o^C for 30 min. The precipitate was extracted by suction-forced filtration, dissolved in distilled water. The solution was dialyzed in MWCO=3500 dialysis bag for 2 days. DLQ-Cy5 conjugates were obtained by lyophilization.

### Preparation and characterization of DLQ/DZ nanocomposites

DLQ lyophilized product (18 mg) was dissolved in distilled water (3 mL) and slowly added 1.2 mL of DOX (5 mg/mL) solution (in DMF). The mixture was ultrasonicated for 30 min in an ice-filled bath. Following this, the resulting solution was dialyzed for 12 h and filtered through 0.8 μm filter to obtain the DLQ/DOX nanocomposites. Additionally, DLQ lyophilized product (18 mg) was dissolved in distilled water (3 mL) and slowly added 1.0 mL of ZnPc (3 mg/mL) solution (in NMP). The mixture was ultrasonicated for 30 min in an ice-filled bath. Afterwards, the resulting solution was dialyzed for 12 h and filtered through 0.8 μm filter to obtain the DLQ/ZnPc nanocomposites. Finally, DLQ lyophilized product (18 mg) was dissolved in distilled water (3 mL) and slowly added 1.2 mL of DOX (5 mg/mL) solution (in DMF) and 1.0 mL of ZnPc (3 mg/mL) solution (in NMP). The mixture was ultrasonicated for 30 min in an ice-filled bath. Following this, the resulting solution was dialyzed for 12 h and filtered through 0.8 μm filter to obtain the DLQ/DZ nanocomposites. To evaluate stability of drug-loaded nanocomposites, the particle size of drug-loaded nanocomposites (DLQ/DOX, DLQ/ZnPc and DLQ/DZ) was measured at 0, 0.5, 1, 2, 3, 4, 5, 6, 8, 10, 12, 24, 36, 48, 72, 96, and 120 h.

### *In vitro* drug release profile

The kinetics of DOX release from DLQ/DZ nanocomposites was investigated using dialysis method *in vitro*. The dialysis bags containing equal amount DLQ/DZ nanocomposites (1 mg DOX) were placed in 200 mL PBS solution (pH 7.4, pH 5.8 or pH 4.5) and shaken at 100 rpm in a horizontal shaker (37 ^o^C). Medium (1 mL) with released drugs was collected at different time points (0, 0.5, 1, 1.5, 2, 3, 4, 5, 6, 7, 8, 10, 12, 24, 36, 48, 72 h), then, 1 mL PBS was added.

### *In vitro* photothermal conversion effect

The thermal profiles of DLQ/DZ in water were measured using 808 nm laser irradiation (FC-808-3000-MM, Changchun Ruilei Optoelectronics Technology Co., Ltd, China) at different power intensity (1, 2 or 3 W/cm^2^) at different concentrations (25, 50, 100, 200 μg/mL of ZnPc) for 10 min. The thermal profiles of ZnPc, DLQ/ZnPc, DLQ/DOX and DLQ/DZ were investigated using 808 nm laser irradiation (2 W/cm^2^ for 10 min) with 200 μg/mL of ZnPc.

### *In vitro* click efficiency and cellular uptake

To evaluate click efficiency, MCF-7 breast cancer cells were incubated in confocal dish at 3×10^4^ cells/dish for 24 h. Following this, MCF-7 cells were treated with 50 μM Ac4ManNAz or serum-free medium for 3 days, respectively. Afterwards, MCF-7 cells were incubated with 20 μM DBCO-Cy5 for 2 h, observed using confocal laser scanning microscopy (LSM700, Carl Zeiss), and analyzed using flow cytometry (BD Accuri C6, USA). Furthermore, MCF-7 cells were seeded in confocal dishes at 3×10^4^ cells/dish and left to grow for 24 h. Afterwards, MCF-7 cells were treated with 50 μM Ac4ManNAz or serum-free medium for 3 days, respectively. Cultured in serum-free medium cells were treated with DLQ-Cy5 medium solution and used as control. The other cells were prepared *via* replacing Ac4ManNAz medium with DBCO-Cy5 or DLQ-Cy5 medium solutions. Afterwards, the cells cultured for 0.5, 1, 2 or 4 h. To visualize cell nuclei, each dish was washed 3 times with PBS and mixed with Hoechst 33342 for 20 min. Following this, the cells were fixed with 4% paraformaldehyde solution for 20 min and observed using the confocal laser scanning microscopy.

To assess cellular uptake, MCF-7 cells were seeded in confocal dishes at 3×10^4^ cells/dish and left to grow for 24 h. Afterwards, the MCF-7 cells were treated with 50 μM Ac4ManNAz or serum-free medium for 3 days, respectively. Following this, DOX, ZnPc, or DLQ/DZ solutions were added to the cells cultured in serum-free medium and left to grow for 6 h and 24 h, respectively. A+DLQ/DZ group was prepared by replacing Ac4ManNAz medium with DLQ/DZ medium for 6 h and 24 h. Additionally, tris (2-carboxyethyl)-phosphine (TCEP) was added for 10 min before adding DLQ/DZ as competitive inhibition group. To visualize cell nuclei, each dish was washed 3 times with PBS and mixed with Hoechst 33342 for 20 min. Afterwards, the cells were fixed with 4% paraformaldehyde solution for 20 min and observed using the confocal laser scanning microscopy (Leica TCS SP5, Germany).

### *In vivo* imaging study

Dynamic distribution of DLQ-Cy5 and DLQ/DZ nanocomposites *in vivo* was monitored using real time imaging. To model MCF-7 breast tumor-bearing nude mice, MCF-7 cell suspension (200 μL; 1×10^7^ cells/mL) was subcutaneously injected into the female nude mice. The mice, bearing expected tumor volume (~500 mm^3^), were randomly divided into 6 groups (3 mice/group): free Cy5, A+DBCO-Cy5, A+DLQ-Cy5, DLQ/DZ, A+DLQ/DZ, and A+TCEP+DLQ/DZ. The A-containing groups were injected intratumorally with Ac4ManNAz (40 mg/kg) once a day for three days. Following this, the mice were administered with Cy5, DBCO-Cy5, DLQ-Cy5 or DLQ/DZ using intravenous injections, respectively. The Cy5 dose was 300 μg/kg. The ZnPc dose was 2 mg/kg. To quench azide groups, competitive inhibition group was intravenously injected with a large amount of TCEP (10 mM) and kept for 1 h. Afterwards, DLQ/DZ solution was injected intravenously. Fluorescence images were observed and photographed at 2, 4, 8, 12, and 24 h after administration. The tumor-bearing nude mice were euthanized after 24 h of administration. All major organs (including heart, liver, spleen, lung, kidney) and tumor tissues were dissected. Finally, each tissue fluorescence image was photographed and semi-quantitatively analyzed. All experiments with animals were approved by the Institutional Animal Care and Use Committee of China Pharmaceutical University.

### *In vivo* temperature measurement

The photothermal conversion capability of DLQ/DZ nanocomposites was investigated in MCF-7 tumor-bearing nude mice using infrared thermal camera (E5, FLIR). The tumor-bearing mice (tumor volume ~200 mm^3^) were randomly divided into 3 groups (3 mice/group): saline, DLQ/DZ, and A+DLQ/DZ. Mice in A+DLQ/DZ group were injected intratumorally with Ac4ManNAz (40 mg/kg) once a day for three days. Afterwards, saline or DLQ/DZ (5 mg ZnPc/kg) were injected intravenously and left for 24 h. To anesthetize, mice were intraperitoneally injected with chloral hydrate (2 mg/kg). Afterwards, the tumor sites of tumor-bearing mice were irradiated at 2 W/cm^2^ for 5 min. The thermographic images were determined using infrared thermal camera.

### *In vitro* cytotoxicity

To determine the cell cytotoxicity of DLQ/DZ nanocomposites, methyl thiazolyl tetrazolium (MTT, 3-(4,5-dimethylthiazol-2-yl)-2,5-diphenyltetrazolium bromide) assay was performed. MCF-7 cells were seeded in 96-well plates at 5000 cells/well, kept for 24 h, and divided into 11 groups: 1. ZnPc; 2. ZnPc+Laser; 3. DOX; 4. DOX+ZnPc+Laser; 5. DLQ/ZnPc; 6. DLQ/ZnPc+Laser; 7. DLQ/DOX; 8. DLQ/DZ+Laser; 9. A+DLQ/DZ+Laser; 10. DLQ; and 11. Ac4ManNAz. The A-containing groups were pre-incubated with Ac4ManNAz (50 μM) for 3 days. Afterwards, cells were incubated with different formulations. Following 4 h-incubation, laser group cells were irradiated for 5 min using 808 nm laser (2 W/cm^2^). Following 24 h-incubation, all cells were added 40 μL MTT (2.5 mg/mL) and left for 4 h. Afterwards, 150 μL DMSO was used to replace medium and dissolve formazan crystals. Absorbance was detected using a microplate reader (POLARstar Omega, BMG LABTECH) at 490 nm. The cell viability was calculated according to the following formula:

Cell viability (%) = (A_test_ - A_blank_) / (A_control_ - A_blank_) × 100%

A_test_, A_control,_ and A_blank_ indicate measured absorbances for test (with formulation added), control (without formulations), and blank (without cells and formulations) groups, respectively.

### *In vivo* anti-tumor efficacy test

Tumor-bearing mice (tumor volume ~50 mm^3^) were randomly divided into 6 groups (5 mice/group): 1. Saline; 2. DOX; 3. DLQ/DOX; 4. DLQ/ZnPc; 5. DLQ/DZ; and 6. A+DLQ/DZ. The A-containing group was injected intratumorally with Ac4ManNAz (40 mg/kg) once a day for three days. The doses of DOX and ZnPc were 5 mg/kg. The formulations were administered using tail vein injection on days 0, 3, and 6. The 4, 5 and 6 groups were irradiated with 808 nm near-infrared light for 5 min (2 W/cm^2^) at days 1, 4, and 7. Tumor volumes were measured daily and calculated using the following formula: (A indicates the tumor width and B indicates the tumor length).

V = (A^2^ × B) / 2

Mice were euthanized on day 20 after injections. Extracted organs (heart, liver, spleen, lung, kidney) and tumors were fixed in 10% (v/v) formalin for immunohistochemistry analysis. Afterwards, the tumor tissues were washed and embedded in paraffin. Sectioned and mounted on slide tissues were stained using H&E and Ki67 immunohistochemical protocols. Slides were observed under an inverted microscope and photographed. The tumor inhibition rate (IR) was calculated according to the following formula: (W_saline_ indicates the average tumor weight of the saline group and W_sample_ indicates the average tumor weight of the formulation groups).

IR (%) = (W_saline_ - W_sample_) / W_saline_ × 100%

### Evaluations of potential side effects associated with drug administration

The body weight of each tumor-bearing mice was recorded daily. Body weight changes were evaluated for signs of systemic toxicity. Additionally, AST (aspartate transaminase), ALT (alanine aminotransferase), and CK (creatine kinase) values in serum of tumor-bearing mice were measured to assess liver and heart toxicity after 20 day-treatment.

### Statistical analysis

Data were given as mean ± standard deviation (SD). Statistical significance was analyzed by one-way unweighted mean analysis of variance (ANOVA) and statistical significance was set at P < 0.05.

## Results and Discussion

### Synthesis and characterization of DLQ conjugate

In this study, the amphiphilic DLQ conjugates were synthesized by a two-step reaction as shown in Figure S8. LMWH, a substance with many carboxyl groups 44, was conjugated with DBCO and Qu by acylation or esterification reactions, respectively. The chemical structure of DLQ conjugates (shown in yellow color, Figure S9) was verified using ^1^H-NMR and FT-IR. In the ^1^H-NMR spectra (Figure S10), the characteristic peak of LMWH was detected at 5.1 ppm and the peaks of DBCO appeared at 1.8 ppm, 5.2 ppm and 7.0-8.0 ppm in the DL spectra. The shown peaks of 8.2 and 6.0-7.0 ppm belonged to Qu, suggesting the formation of DLQ conjugates. In the FT-IR spectra of LMWH (Figure S11), the absorption peaks at 3468.6 cm^-1^ and 1627.6 cm^-1^ were attributed to hydroxyl and carbonyl stretching vibrations of LMWH, respectively. Compared to LMWH spectra, the new absorption bands at 1682.8 cm^-1^ and 1563.0 cm^-1^ were ascribed to I (C=O stretching vibration) and II (N-H bending vibration) bands of amide bond in DL spectra, respectively, indicating that LMWH and DBCO was conjugated by amide bonds. Furthermore, characteristic absorption bands of ester bonds (1730.6 cm^-1^) and amide bonds (1684.2 cm^-1^ and 1567.7 cm^-1^) were observed in DLQ spectra, confirming the successful synthesis of DLQ conjugates. The molar grafting ratios of DBCO and Qu in DLQ conjugates were 29.8% and 15.0%, respectively (Figure S12). The critical aggregation concentration (CAC) of the DLQ was 77.76 μg/mL (Figure S13), indicating that DLQ conjugation would self-assemble into nano-sized system at a low concentration and possess excellent dilution stability 45.

### Preparation and characterization of DLQ/DZ nanocomposites

**Figure 1:**
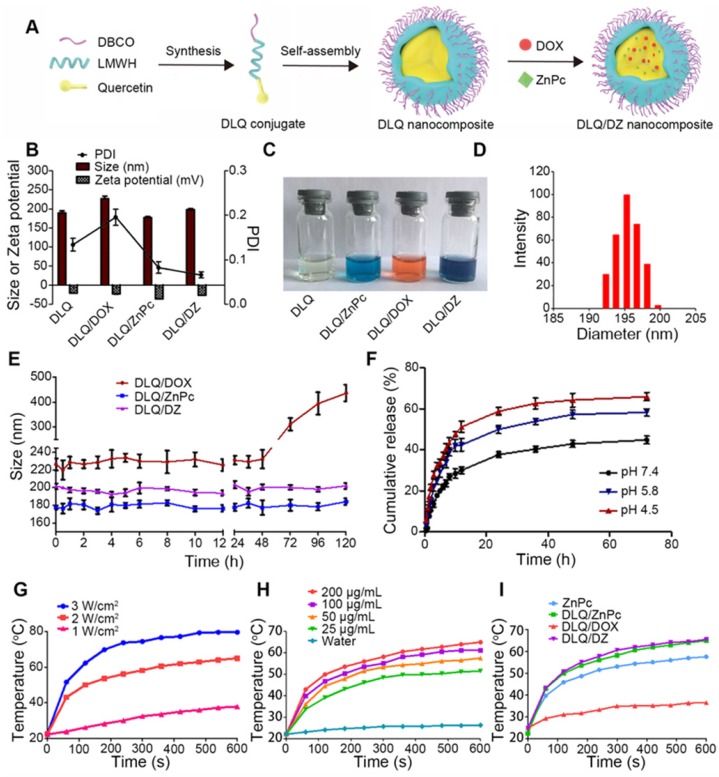
(A) Synthesis and assembly process of DLQ/DZ nanocomposites. (B) The polydispersity index (PDI), particle size and zeta potential of DLQ, DLQ/DOX, DLQ/ZnPc, and DLQ/DZ nanocomposites. (C) Photographs of DLQ, DLQ/ZnPc, DLQ/DOX, and DLQ/DZ nanocomposites in distilled water. (D) The DLS image of DLQ/DZ nanocomposites. (E) The stability of DLQ/DOX, DLQ/ZnPc, and DLQ/DZ nanocomposites in distilled water for 120 h. (F) *In vitro* release of DOX from DLQ/DZ nanocomposites at pH 7.4, pH 5.8 and pH 4.5 at 37 ^o^C. (G) The temperature elevation profiles of DLQ/DZ nanocomposites in different laser power (1, 2 and 3 W/cm^2^) for 600 s. (H) The temperature elevation profiles of DLQ/DZ nanocomposites at different ZnPc concentration (25, 50, 100 and 200 μg/mL). (I) The temperature elevation profiles of ZnPc, DLQ/ZnPc, DLQ/DOX, and DLQ/DZ upon 808 nm laser irradiation at 2 W/cm^2^.

The DBCO-modified, co-incorporating DOX and ZnPc (DLQ/DZ) nanocomposites were prepared using dialysis method (Figure 1A). Two single drug loaded nanocomposites (i.e. DLQ/DOX and DLQ/ZnPc) were also prepared as controls. As shown in Figure 1B, 1C and 1D, the particle size of dual-loading DLQ/DZ nanocomposites was 198.2±2.4 nm (shown in dark blue color with a spherical shape in Figure S14). Other control nanocomposites demonstrated similar particle size distributions (shown in different colors). The nano-scaled particle of this size demonstrated unique tumor tissue-specific permeability and retention 46. Additionally, these formulations were negatively charged (> 20 mV), able to avoid plasma protein adsorption to a great extent, and prolong time of nanocomposites circulation in blood 47, 48.

Surprisingly, DOX- and ZnPc-loading levels in DLQ/DZ nanocomposites were approximately 10%, *i.e.* up to about 20% of total drug loading was achieved; while the drug loadings in DLQ/DOX and DLQ/ZnPc nanocomposites were 10.6±3.2% and 11.2±2.1% respectively. It was indicated that drug co-loading increased total drug loading capacity of DLQ nanocomposites, which made DLQ/DZ nanocomposites an optimal nanosystem for combination therapy. The joint effect of hydrophobic and π-π stacking interactions among DOX, ZnPc and Qu might be the driving force of elevated drug loading. This also appeared to have improved the stability of nano-system as shown in Figure 1E. The particle sizes of DLQ/DOX nanocomposites remained relatively stable in water only within 48 h; while no significant size changes were observed for DLQ/DZ and DLQ/ZnPc nanocomposites during over 120 h.

Furthermore, the *in vitro* release behavior of DOX from nanocomposites was evaluated in PBS buffer at pH 7.4, 5.8 and 4.5. As shown in Figure 1F, the cumulative amount of released DOX was lower at pH 7.4 compared to pH 5.8 and 4.5. The effects might be associated with destruction of electrostatic interactions between drug molecules and DLQ under the acid pH conditions 15, 49, 50. The observed drug-release property favored the agent therapeutic effect in the acidic microenvironment of tumors.

### The photo-thermal conversion capability of DLQ/DZ nanocomposites

Previous studies demonstrated that temperature above 48 ^o^C effectively kills tumor cells 51, *i.e.* the temperature increments (ΔT) > 18 ^o^C (*via* the subcutaneous tumor temperature of ~30 ^o^C) were believed to induce necrosis in tumor cells. The photo-thermal conversion capability of DLQ/DZ nanocomposites was investigated using laser irradiation. As shown in Figure 1G, the temperature of DLQ/DZ solution was gradually increased along with the increases in irradiation time and power density. We found that the ΔT of DLQ/DZ solution irradiated at 2 W/cm^2^ and 3 W/cm^2^ rapidly went up to 20.5 ^o^C and 28.7 ^o^C during 60 s, respectively. These results revealed the favorable potential of laser power to kill tumor cells at 2 W/cm^2^ or 3 W/cm^2^. Additionally, irradiating at 2 W/cm^2^ in DLQ/DZ solution (concentration range of 25-200 μg/mL) for 600 s forced ΔT to rise from 29.3 to 42.8 ^o^C (Figure 1H); while using distilled water (as control) resulted in negligible temperature changes (< 5 ^o^C) under equal conditions. These results indicated that the thermal effect induced by DLQ/DZ nanocomposites strongly depended on power density and ZnPc concentration. It was noted that the ability of DLQ/DZ and DLQ/ZnPc nanocomposites to convert photo energy into thermal energy was much higher than the ability of free ZnPc solution (Figure 1I), indicating that the encapsulation of ZnPc into DLQ nanocomposites favored their photothermal conversion. Consequently, the photothermal profiles of DLQ/DZ and DLQ/ZnPc nanocomposites were similar during the irradiation; while the DLQ/DOX solution showed only a slight temperature elevation due to light exposure. Furthermore, the photothermal conversion efficiency of DLQ/DZ nanocomposites exposed to 808 nm continuous laser was 21.9% (Figure S15-S16). The photothermal performance remained stable after four irradiation natural cooling cycles (Figure S17). In brief, DLQ/DZ nanocomposites possessed high photothermal conversion efficiency, which could favor photothermal therapy *in vivo*.

### Generation of azide groups and *in vitro* click reaction efficiency

**Figure 2:**
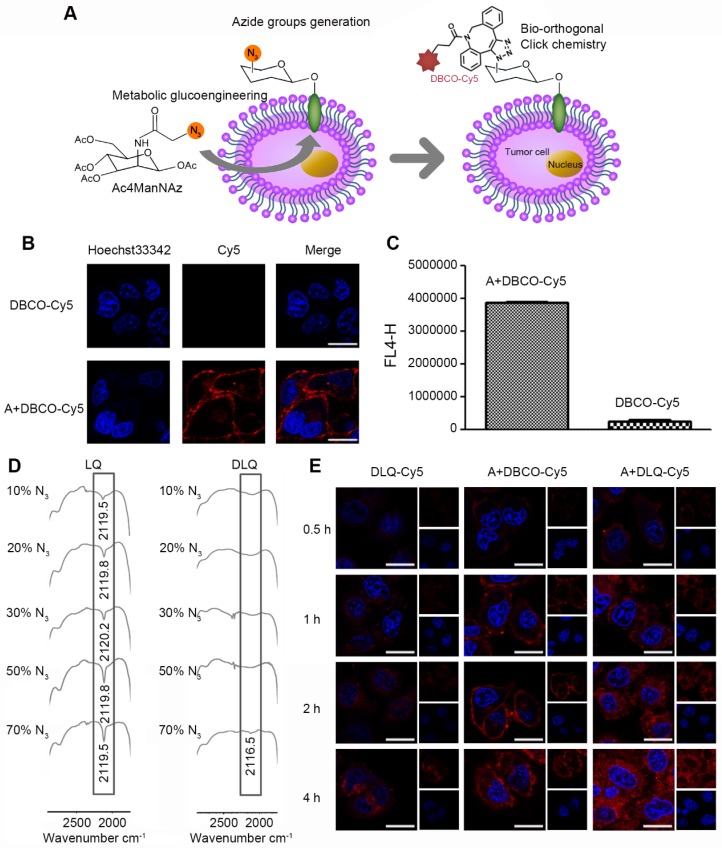
(A) Schematic illustration of tumor cell-targeting strategy by metabolic glycoengineering and bio-orthogonal click chemistry. (B) Visualization of azide groups (red) on the surface of Ac4ManNAz-treated MCF-7 cells, and the cells without Ac4ManNAz-treated were used as a control. Scale bar is 20 μm for all images. (C) Mean fluorescence intensity detected by flow cytometry in MCF-7 cells after incubation with DBCO-Cy5 for 2 h. (A: Ac4ManNAz pre-treated MCF-7 cells). (D) FT-IR spectra of LQ or DLQ nanocomposites with different percentages of azide groups (10%, 20%, 30%, 50% and 70%). (E) Cellular binding and uptake for 0.5 h, 1 h, 2 h and 4 h after incubation with DLQ-Cy5, A+DBCO-Cy5, A+DLQ-Cy5. Scale bar is 20 μm.

The tumor cells and nanocomposites click efficiency is a key component of tumor-targeted therapy 36. In this case, it was essential that metabolic precursor (Ac4ManNAz) firstly produced abundant non-natural azide groups on the surface of MCF-7 tumor cells through intracellular metabolic glycoengineering 35. To detect the azide groups on the membrane of tumor cells, DBCO-Cy5 was chosen because it could specifically bound to the azide groups *via* bio-orthogonal copper-free click chemistry reaction 33 and could be directly visualized by the spontaneous red fluorescence (Figure 2A). The number of azide groups on the surface of tumor cells was calculated to be 3.23×10^7^/cell through determining the fluorescence intensity of the MCF-7 cells pretreated with Ac4ManNAz after incubation with DBCO-Cy5 for 1 h (Figure S18). The intensity was significantly higher than that of naturally expressed receptors (e.g. laminin-binding intensity (10^3^-10^5^/cell) reported in previous studies 37, 52). In other words, the number of the targetable receptors was enhanced by 2-4 orders of magnitude in our case. The large number of azide groups produced on cell membranes provided more opportunities and higher probability for subsequent initiation of cell-level click chemistry reactions.

Furthermore, click reaction efficiency of DBCO and azide groups was investigated by confocal imaging, flow cytometry, and FT-IR analysis* in vitro*. As shown in Figure 2B, the A+DBCO-Cy5 group generated strong red fluorescence signal at MCF-7 cell membranes after incubation for 2 h; while the DBCO-Cy5 group without pretreatment with Ac4ManNAz showed no obvious red fluorescence. The quantitative analysis demonstrated that the fluorescence intensity of the A+DBCO-Cy5 group was significantly higher than that of the DBCO-Cy5 group (almost 9 times) (Figure 2C). The observed increase was attributed to the effective generation of azide groups on the surface of MCF-7 cells. The excellent click reaction efficiency of DBCO and azide groups on the surface of tumor cells demonstrated successful combination of metabolic glycoengineering with bio-orthogonal copper-free click chemistry, which may lead to high potential in tumor-targeted drug delivery.

The click chemistry reaction efficiency of the DLQ nanocomposites and azide groups was measured using the absorption peak of azide groups (around 2100 cm^-1^) within FT-IR spectrum 2 h after initiation of the reaction. The LQ nanocomposites without DBCO was used as the control. As shown in Figure 2D, in the LQ group, along with the increasing quantity of azide groups incubated, the azide group absorption peak became sharper. On the contrary, when 10%-50% of azide groups were exposed to DLQ, the absorption peak completely disappeared, indicating that all azide groups completely reacted with DLQ nanocomposites in water. However, some of the azide groups did not react when their amount reached 70% (represented by a tiny peak, Figure 2D). Meanwhile, when 50% azide groups were exposed to the DLQ in serum or tumor homogenate, the azide groups completely reacted with DLQ nanocomposites (Figure S19). These results indicated that at least 50% of azide groups were specifically clicked to DBCO on the DLQ within 2 h, suggesting that the click chemistry reaction was fast and highly efficient *in vitro* and* in vivo*.

The intracellular fate of DBCO-based nanocomposites was monitored by grafting of the fluorescent dye Cy5 into the DLQ conjugates. Subsequently, we investigated the time course of click reaction on the surface of MCF-7 cells and associated intracellular processing of DLQ-Cy5 nanocomposites using confocal laser scanning microscopy. As shown in Figure 2E, the fluorescence intensity in all experimental groups gradually increased over time, suggesting that the cellular uptake was time-dependent. Analyzing time-lapse imaging, we found that DBCO-Cy5 fluorescence in Ac4ManNAz-pretreated cells mainly appeared on the cell membranes at different time points. The data indicated the occurrence of click reactions based on DBCO and azide groups on tumor cell membranes. However, low level of fluorescence was detected in the cytoplasm during 4 h of A+DBCO-Cy5 treatment. The red fluorescence of DLQ-Cy5 in Ac4ManNAz-pretreated cells was observed on the cell membrane as early as 0.5 h, and was then successively taken up into the cells since 1 h. These results suggested that DLQ-Cy5 nanocomposites were first bound to the tumor cells by click chemical reactions and then entered into the cells *via* endogenous glycan internalization and endocytosis 33. Furthermore, the cell cytoplasm in the A+DLQ-Cy5 group was marked by strong red fluorescent signal at 4 h. The effects were also significantly higher than that in the DLQ-Cy5 group, revealing the effective and fast tumor cellular uptake was mainly based on specific reactions between the DBCO on DLQ nanocomposites and the azide groups on the surface of tumor cells. Interestingly, DLQ-Cy5 nanocomposites exhibited significantly different intracellular distribution and efficiency compared with DBCO-Cy5, which was attributed to the polyvalent interactions 53-55 of nanocomposites. In other words, multiple DBCO on the nanocomposites can simultaneously bind to multiple azide groups on the cell surface compared to the monovalent interaction of single molecules. Thus, the superiority of DBCO-based nanocomposites can be used to improve tumor-targeted drug delivery. Taken together, DLQ nanocomposites with the capacity of efficient clicking with the azide groups generated by tumor cells offered the prerequisite for effective drugs uptake by tumor cells.

### Cellular uptake and biodistribution of DLQ/DZ nanocomposites

**Figure 3:**
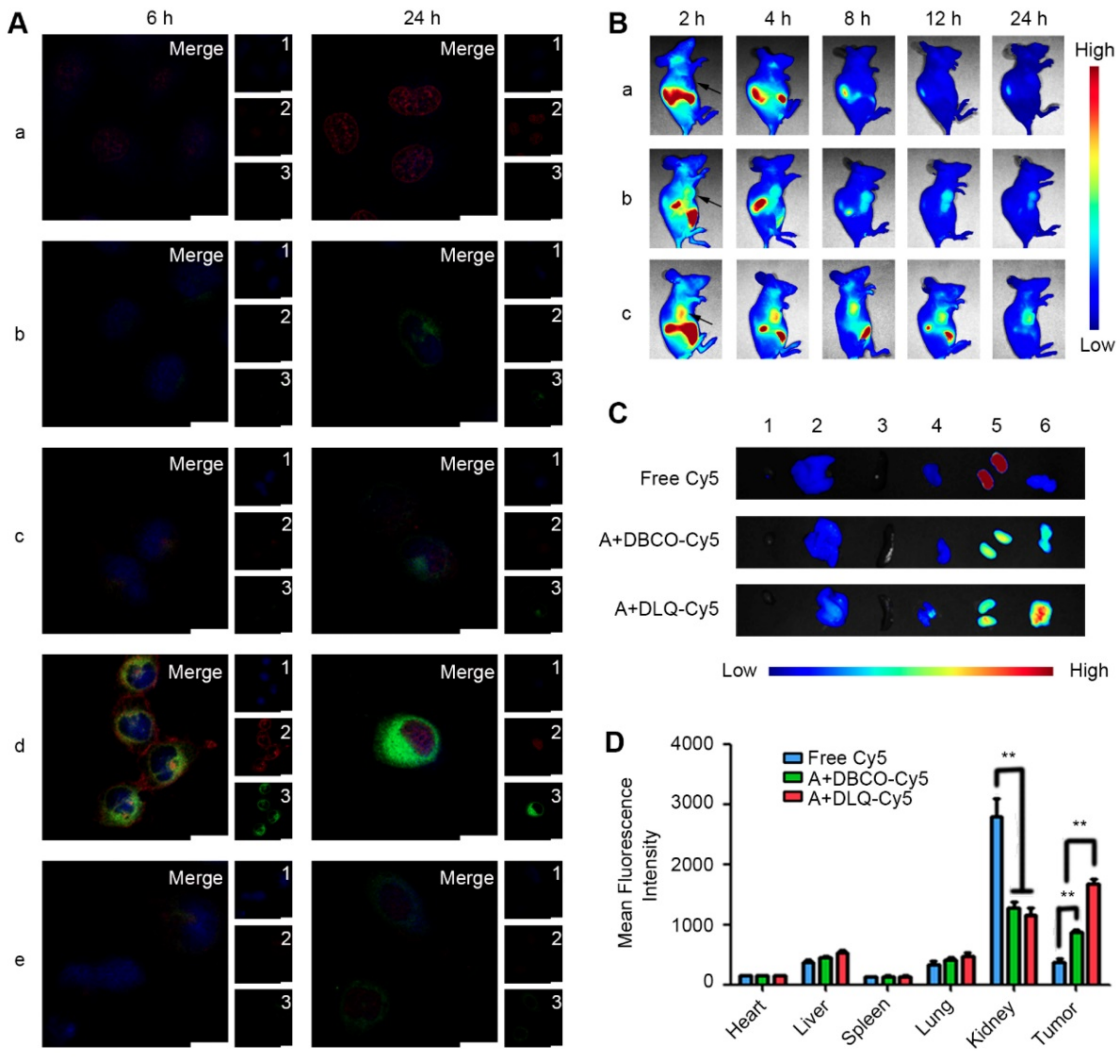
(A) Time-dependent cellular uptake after 6 h and 24 h incubation with DOX (a), ZnPc (b), DLQ/DZ (c), A+DLQ/DZ (d), and A+TCEP+DLQ/DZ (e) in MCF-7 cells. 1: Nucleus (blue); 2: DOX (red); 3: ZnPc (green); Merge: overlay of 1, 2 and 3. Scale bar is 20 μm. (B)* In vivo* NIR fluorescence images of MCF-7 tumor-bearing nude mice at 2 h, 4 h, 8 h, 12 h, and 24 h following injection free Cy5 (a), A+DBCO-Cy5 (b) and A+DLQ-Cy5 (c). Arrows indicated the tumor site. (C) *Ex vivo* NIR fluorescence images of hearts (1), livers (2), spleens (3), lungs (4), kidneys (5), and tumors (6) at 24 h post-injection. (D) Quantitative analysis of mean fluorescence intensity in normal tissues and tumors at 24 h post-injection. Data are represented as mean±SD (n=3). ^**^P < 0.01.

To validate the efficient and prominent tumor-targeting ability of DBCO-modified nanocomposites, we investigated the nanocomposites cell-uptake *in vitro* and biodistribution of DLQ/DZ nanocomposites *in vivo* using confocal laser scanning microscopy and non-invasive optical imaging system, respectively. As shown in Figure 3A, the red (DOX) and green (ZnPc) fluorescence intensities in A+DLQ/DZ group were notably higher than those in the DLQ/DZ group, suggesting that bio-orthogonal click reactions on the surface of tumor cells effectively increased the cellular uptake of DLQ/DZ nanocomposites. This was also supported by the results observed in the A+TCEP+DLQ/DZ group. The fluorescence signals of DOX and ZnPc were dramatically weakened after treatment with azide-quenching agent TCEP. The red fluorescence of DOX in the A+DLQ/DZ group was observed in the cytoplasm and cell membrane at 6 h; while there was no significant fluorescent signal on the cell membrane in the DLQ/DZ group without pre-treatment with Ac4ManNAz, further supporting that the cellular uptake of DLQ/DZ nanocomposites relied on binding to azide groups on the cell surface. Notably, the green fluorescence of ZnPc in the A+DLQ/DZ group was localized in the cytoplasm rather than retained at the cell surface at 6 h, which could be associated with the self-quenching effect of ZnPc encapsulated in nanocomposites 56, 57. The quenched ZnPc fluorescent signals were recovered once the encapsulated ZnPc was released after cell internalization contributing to stronger green fluorescent signals in cytoplasm. After incubation of the Ac4ManNAz-pretreated MCF-7 cells with DLQ/DZ for 24 h, DOX was mostly observed in the cell nuclei; while ZnPc was homogeneous distributed in the cytoplasm. The suitable intracellular distribution would help chemotherapy drug and photothermal conversion agent to achieve better therapeutic effects.

Non-invasive optical imaging was used to track the* in vivo* distribution of DLQ nanocomposites (labeled covalently with Cy5). As shown in Figure 3B and 3C, the A+DLQ-Cy5 group was marked by stronger and longer-lasting in-tumor fluorescence compared to that in the A+DBCO-Cy5 and free Cy5 groups. The in-tumor fluorescent intensity was 1.9-times and 4.6-times higher than that in the A+DBCO-Cy5 and free Cy5 groups, respectively. Moreover, obviously decreased distribution of the dye in kidneys was obtained in two other groups compared with free Cy5 group (P < 0.01) (Figure 3D). These results indicated the important role of DBCO-mediated bio-orthogonal click reaction in improving their biodistribution* in vivo*. Such distribution could also reduce potential adverse effects. Meanwhile, tumor-targeting nanocomposites (A+DLQ-Cy5) were preferable materials for anti-tumor drug delivery compared to small molecules (A+DBCO-Cy5) 53-55.

**Figure 4:**
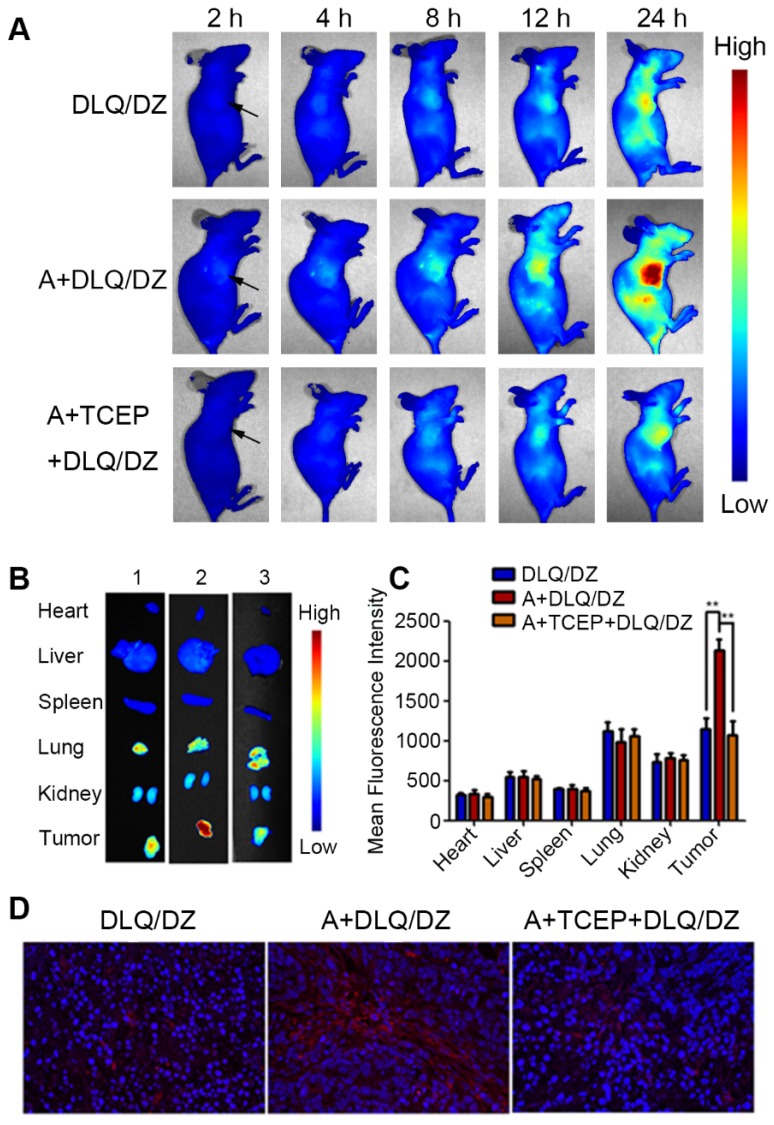
(A)* In vivo* fluorescence images of MCF-7 tumor-bearing nude mice at 2 h, 4 h, 8 h, 12 h and 24 h following injection DLQ/DZ, A+DLQ/DZ and A+TCEP+DLQ/DZ. Arrows indicate the tumor site. (B) *Ex vivo* fluorescence images of heart, liver, spleen, lung, kidneys and tumor at 24 h post-injection of DLQ/DZ (1), A+DLQ/DZ (2), A+TCEP+DLQ/DZ (3). (C) Quantitative analysis of mean fluorescence intensity in normal tissues and tumors. Data are represented as mean±SD (n=3). ^**^P < 0.01. (D) Fluorescence images (400-fold magnification) of tumor sections. Red was ZnPc and blue (DAPI) was the nucleus.

Besides the selective accumulation of nanocarriers at tumor tissues, opportune drug-release from nanocarriers is of equal importance for anticancer drugs in tumor cells, which is the ultimate goal of effective anti-tumor drug delivery. To study the drug delivery efficiency of DLQ nanocomposites, we further investigated the biodistribution of drugs using fluorescence signals emitted by ZnPc (encapsulated in the DLQ/DZ nanocomposites). As shown in Figure 4, *in vivo* and *ex vivo* tumor fluorescence signals in the A+DLQ/DZ group were higher than those in the DLQ/DZ group. The result was consistent with the *in vitro* results of the cellular uptake experiments (Figure 3A). When the azide groups on the surface of tumor cells were quenched by TCEP as an inhibitor (A+TCEP+DLQ/DZ group), the fluorescence signal within tumor tissues decreased significantly. This illustrated that the generation of azide groups on the surface of the tumor cells was a prerequisite for the bio-orthogonal click chemistry to prompt accumulation of DLQ/DZ nanocomposites at tumor sites* in vivo*. Considering the relatively short residence of empty nanocarriers in tumors (about 12 h, as shown in Figure 3B), we found weak ZnPc fluorescence signals at 2 h, which kept increasing until 24 h (Figure 4A). These results indicated that the drugs were efficiently delivered by DLQ vectors, released into tumor cells, and recovered fluorescence signals. The fluorescence was maintained in tumor for more than 24 h, suggesting a favorable drug accumulation and retention in tumor cells, which was supposed to facilitate the anti-tumor effect of drugs. Furthermore, the level of in-tumor ZnPc in the A+DLQ/DZ group was significantly higher than that in other tissues including liver and spleen (Figure 4B and 4C) and corresponded to the distribution efficiency of empty DLQ carriers *in vivo* (Figure 3D). Comparing the traditional tumor-targeting nanoparticles, including our previous studies 40, 58, the distribution of nanoparticles or drugs in tumor tissue was similar or lower than that in the liver or spleen. Thus, highly selective tumor-targeting achieved in the current study illustrated the superiority and promising prospect of elaborated artificial modification strategy in tumor targeting nanocomposite designation.

### *In vitro* cytotoxicity and *in vivo* photothermal conversion

**Figure 5:**
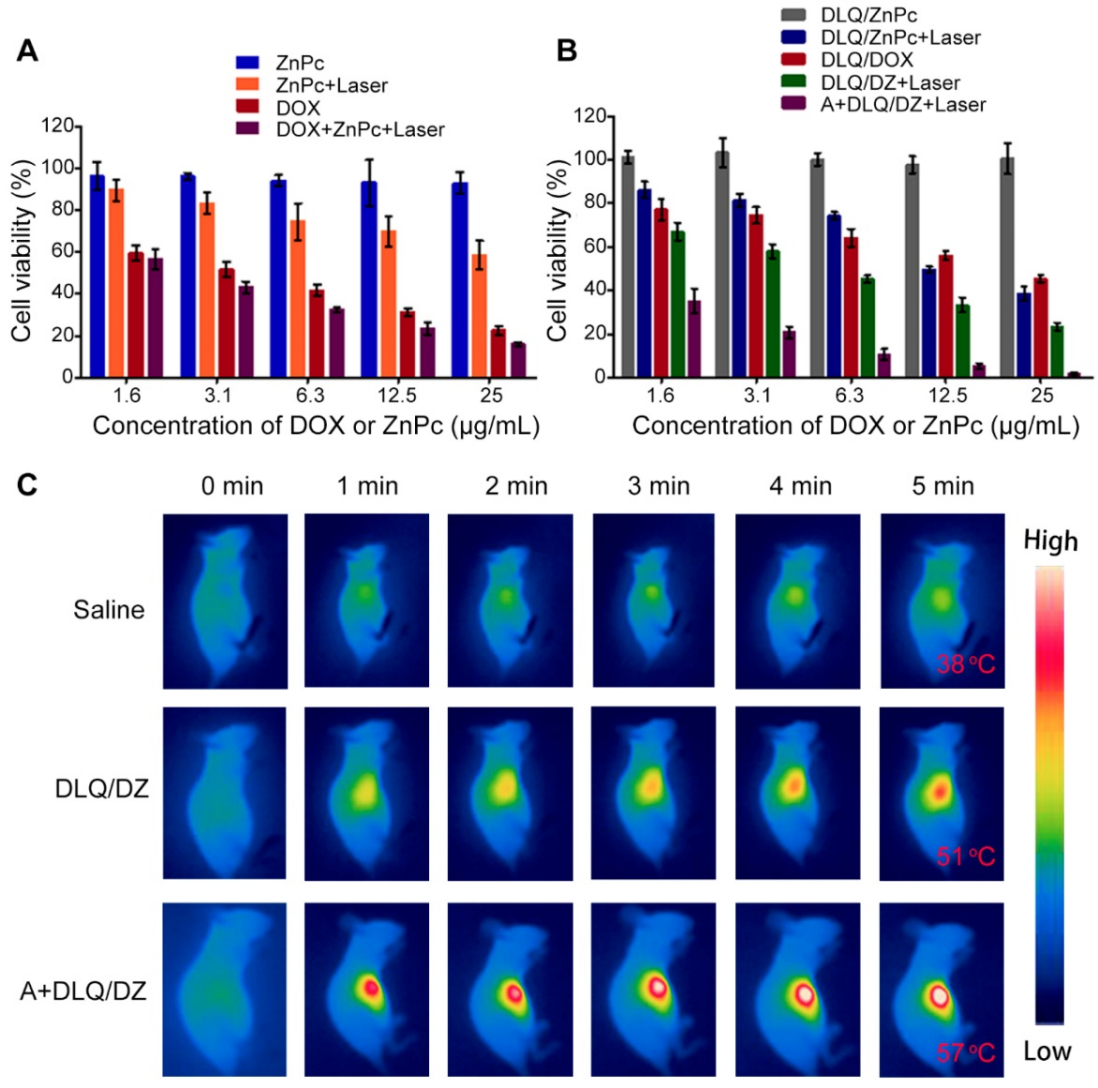
(A) Cytotoxicity of MCF-7 cells treated with ZnPc, ZnPc+Laser, DOX, and DOX+ZnPc+Laser. (B) Cytotoxicity of MCF-7 cells treated with DLQ/ZnPc, DLQ/ZnPc+Laser, DLQ/DOX, DLQ/DZ+Laser, and A+DLQ/DZ+Laser. Data are presented as the mean±SD (n=6). (C) Infrared thermal images of MCF-7 human tumor-bearing nude mice treated with saline, DLQ/DZ, and A+DLQ/DZ during the laser irradiations.

MTT assay and *in vivo* infrared thermography were utilized to assess the effect of the enhanced tumor-targeting delivery on cytotoxicity and photothermal conversion, respectively. The viability of cells treated with Ac4ManNAz and DLQ nanocomposites was above 90% (Figure S20), suggesting their minor cytotoxicity against MCF-7 tumor cells. Additionally, the difference in cell viability between the laser-exposed (ZnPc+Laser and DLQ/ZnPc+Laser) and non-exposed (ZnPc and DLQ/ZnPc) groups indicated that ZnPc had higher NIR cytotoxicity (Figure 5A). Moreover, DOX+ZnPc+Laser group exhibited remarkably enhanced cytotoxic response than that in the DOX or ZnPc+Laser groups alone. Similarly, the cell mortality in the DLQ/DZ+Laser group was significantly higher than that in the DLQ/DOX and DLQ/ZnPc+Laser groups (Figure 5B). Furthermore, the calculated combination index (CI) values of DOX+ZnPc+Laser and DLQ/DZ+Laser using the half maximal inhibitory concentration of agents in MCF-7 cells were 0.6, suggesting a synergistic effect of chemotherapy and PTT. Importantly, a sharp decrease in cell viability was found in the A+DLQ/DZ+Laser group compared to the DLQ/DZ+Laser group. It suggested that the combined strategy of metabolic glycoengineering and click chemistry improved the cytotoxicity of DLQ/DZ nanocomposites.

The infrared thermography results shown that the A+DLQ/DZ treatment promoted the tumor surface temperature in mice *via* increased tumor accumulation of PTT agents. As shown in Figure 5C, laser irradiation (808 nm at 2 W/cm^2^) for 5 min increased the tumor surface temperature in mice in the A+DLQ/DZ-treated group to 57 ^o^C; while in DLQ/DZ-treated group, temperature was increased to 51 ^o^C under the same condition.

### *In vivo* anticancer efficacy

**Figure 6:**
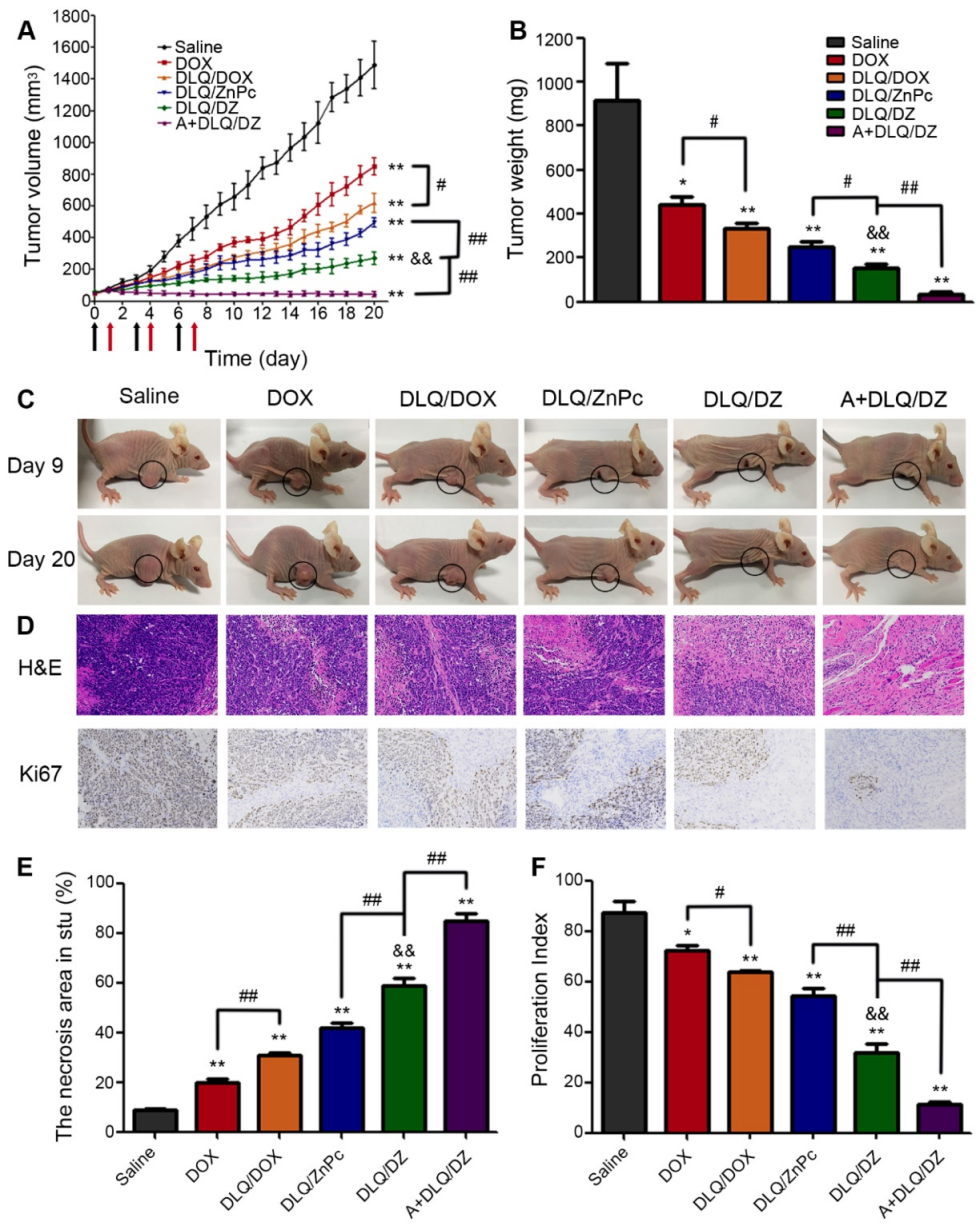
(A) The changes of tumor volume of MCF-7 human tumor-bearing nude mice from saline, DOX, DLQ/DOX, DLQ/ZnPc, DLQ/DZ, and A+DLQ/DZ treatment groups. Black arrows for tail vein administration, red arrows for laser irradiation. (B) Tumor weights of MCF-7 human tumor-bearing nude mice from different treatment groups at day 20. Data are represented as mean±SD (n=5) (C) Representative photographs of MCF-7 human tumor-bearing nude mice from different treatment groups at day 9 and 20. (D) H&E and Ki67 staining images (200-fold magnification) of the tumor tissues from different treatment groups. (E)The necrotic cells percentage of the tumor tissues after treatment. (F) The corresponding proliferating index of the tumor tissues in the Ki67 staining assay. Data are presented as the mean±SD (n=5). ^**^P < 0.01, ^*^P < 0.05 vs. the saline group, ^&&^P < 0.01 vs. the DLQ/DOX group, ^#^P < 0.05,^ ##^P < 0.01.

Encouraged by the bio-distribution and infrared thermal imaging data *in vivo*, we further evaluated the therapeutic potential of DLQ/DZ nanocomposites on MCF-7 human tumor-bearing nude mice. As shown in Figure 6A, the data could be divided into three categories. Firstly, single agent treated groups such as DOX, DLQ/DOX or DLQ/ZnPc groups demonstrated a moderate inhibition of tumor growth (less desirable in cancer therapy). Secondly, in the DLQ/DZ groups, two-component simultaneous delivery of DOX and ZnPc exhibited stronger antitumor effect compared to single agent treated groups (P < 0.01), implying that the two-component combination of chemotherapy and PTT was more effective than monotherapy. However, DLQ/DZ treatment did not completely suppress tumor growth, likely due to insufficient accumulation of nanocomposites in tumor site. Excitingly, the tumor growth of mice after three-component combined treatment (in the A+DLQ/DZ group) was well controlled by the aid of efficient and specific tumor targeting delivery, and the tumor-inhibition rate was up to 96.1%. The effect was better than those observed in the two-component treatment (DLQ/DZ: 83.3%) and the single treatment (DOX: 51.5%, DLQ/DOX: 63.7%, DLQ/ZnPc: 72.7%) groups. Furthermore, it was worth noting that the tumor volume in mice from the A+DLQ/DZ group was not increased during the investigated period, even after the schedule of the drug administration and irradiation (9-20 days) compared to other groups, indicating an excellent and long-lasting inhibition of tumor progression. The tumor weight and appearance (Figure 6B and 6C) also demonstrated a desirable mutually synergistic performance of the therapy in the A+DLQ/DZ group. Immunohistochemistry analysis exhibited larger necrosis area (84.7%) and lower proliferation index (11.3%) in the A+DLQ/DZ group (Figure 6D, 6E, and 6F). These results indicated that A+DLQ/DZ group yielded the highly efficient antitumor effect. The effect was supported by the fast and tumor-specific drug delivery of DLQ/DZ nanocomposites using the bio-orthogonal click-targeting that intensified accumulation of both drugs in tumor cells. The accumulation led to the rapid rise of temperature in tumor tissues (Figure 5C) and contributed to more effective tumor ablation allied with chemotherapeutics (Figure 6A). Meanwhile, the achieved hyperthermia further facilitated the deep tumor penetration of chemotherapeutics by expanding endothelial gaps and enhancing blood pressure and blood flow 18-21, and increased the tumor drug accumulation *via* enhancing vascular permeabilization 22, forming a positive feedback loop.

### *In vivo* potential side effects of nanocomposites

**Figure 7:**
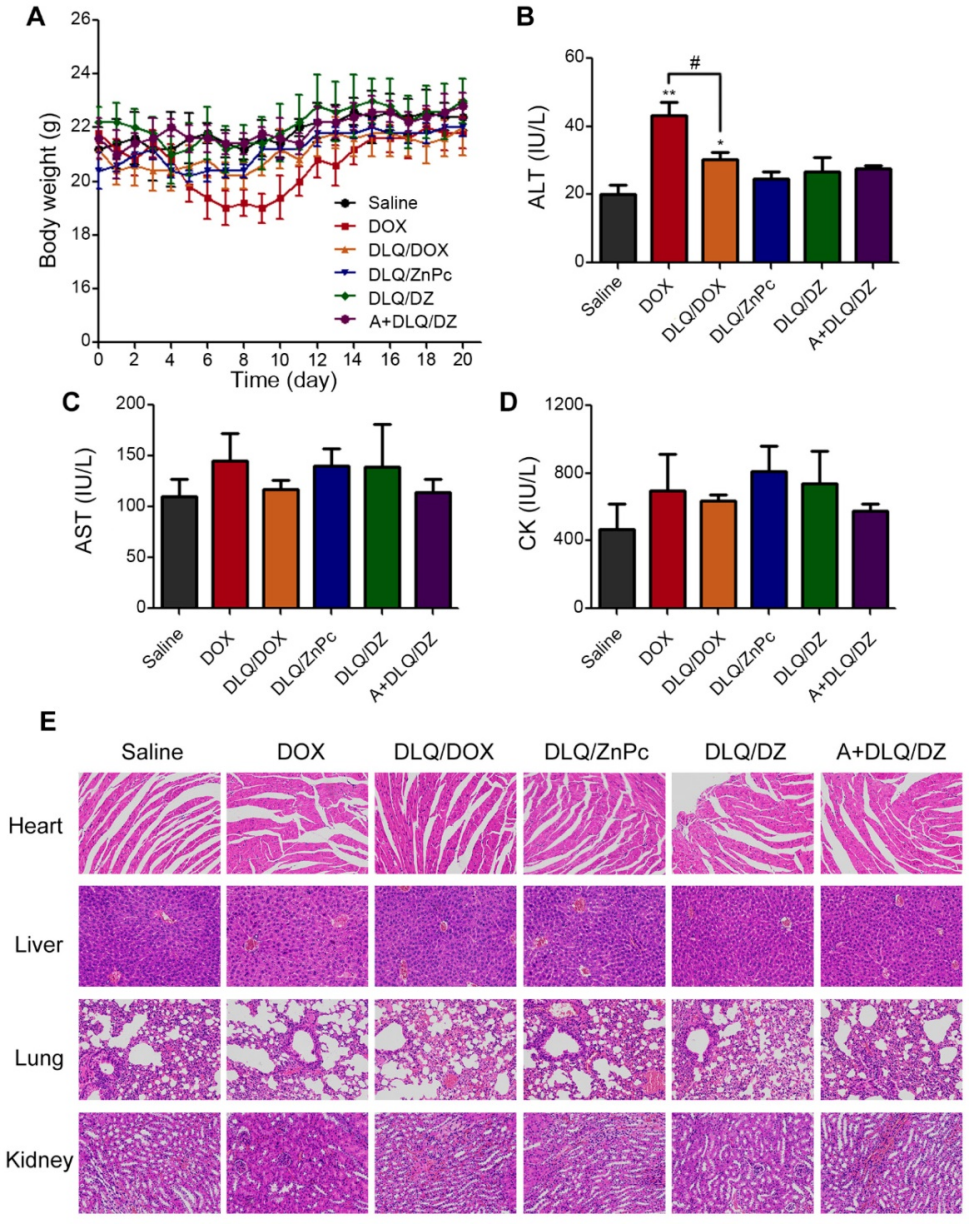
The body weight (A), ALT (B), AST (C) and CK (D) values of MCF-7 human breast tumor-bearing nude mice from saline, DOX, DLQ/DOX, DLQ/ZnPc, DLQ/DZ, and A+DLQ/DZ treatment groups. Data are represented as mean±SD (n=5). ^**^P < 0.01, ^*^P < 0.05 vs. the saline group, ^#^P < 0.05. (E) H&E staining images (200-fold magnification) of the heart, liver, lung, kidney from different treatment groups.

The Figure 6C was the visual therapeutic results in tumor-bearing nude mice after 9 and 20 days of treatment. The A+DLQ/DZ group showed obvious photothermal damage and scar formation at the tumor site on day 9 post-treatment. The scar tissue was shed at day 20 confirming good *in vivo* tolerance to the A+DLQ/DZ nanocomposites. The body weight (Figure 7A) of tumor-bearing nude mice in DOX group decreased significantly on day 9. The ALT value (Figure 7B) increased significantly in this group compared to saline group, indicating that DOX had severe systemic and hepatotoxic effects. In contrast, DLQ/DOX group showed an increase in body weight and a decrease in ALT value (P < 0.05) compared to DOX group, suggesting that DOX encapsulation in nanocomposites could reduce its toxicity. Moreover, the body weight, ALT, AST (Figure 7C), and CK values (Figure 7D) in the DLQ/DZ and the A+DLQ/DZ groups demonstrated no significant differences compared to saline group, indicating their better biosafety characteristics. The superior biosafety of agents in the A+DLQ/DZ group was further confirmed in the histological analysis of the major organ tissues (Figure 7E). Observed results showed no significant side effects in the A+DLQ/DZ group, supporting a high potential of this strategy in future clinical applications.

## Conclusion

In summary, we have successfully fabricated a nanocomposite for targeted chemo-photothermal combination therapy based on metabolic glycoengineering and bio-orthogonal copper-free click chemistry. The nano-system demonstrated superior tumor targeting ability and facilitated the precise co-delivery of both chemotherapeutic agents and photosensitizers. As a result, the orchestrated DLQ/DZ nanocomposites exerted a promising antitumor effect with an acceptable safety profile. This work provides insights into the improvement of cancer therapies, particularly for tumors at anatomically superficial locations, such as breast cancers and melanomas. In addition, as successful combination therapy and efficient drug delivery are equally important for a variety of pathological treatments, the application of the bio-orthogonal click-targeting combination strategy used in this study may be extended beyond tumor treatment.

## Supplementary Material

Supplementary methods and figures.

## Abbreviations

PTT: photothermal therapy
NIR: near-infrared
ZnPc: zinc phthalocyanine
DOX: doxorubicin
LMWH: low molecular weight heparin
Qu: quercetin
DBCO: dibenzocyclooctyne
Ac4ManNAz: tetraacetylated N-azidoacetyl-D-mannosamine
EPR: enhanced permeability and retention
LQ: low molecular weight heparin-quercetin conjugates
DL: dibenzocyclooctyne-low molecular weight heparin conjugates
DLQ: dibenzocyclooctyne-low molecular weight heparin-quercetin conjugates
CAC: critical aggregation concentration
DLQ/DZ: dibenzocyclooctyne-low molecular weight heparin-quercetin nanocomposites co-incorporating DOX and ZnPc
DLQ/DOX: DOX-loaded dibenzocyclooctyne-low molecular weight heparin-quercetin nanocomposites
DLQ/ZnPc: ZnPc-loaded dibenzocyclooctyne-low molecular weight heparin-quercetin nanocomposites
DLS: dynamic light scattering
TEM: transmission electron microscopy
DBCO-Cy5: Cy5-labeled dibenzocyclooctyne
DLQ-Cy5: Cy5-labeled dibenzocyclooctyne-low molecular weight heparin-quercetin nanocomposites
TCEP: tris (2-carboxyethyl)-phosphine
MTT: methyl thiazolyl tetrazolium
CI: combination index
ALT: alanine aminotransferase
AST: aspartate transaminase
CK: creatine kinase
NMP: N-methylpyrrolidone
EDC: 1-Ethyl-3 (3-dimethylaminopropyl) carbodiimide
NHS: N-hydroxysuccinimide
DMF: dimethylformamide
IR: tumor inhibition rate
SD: standard deviation
