# Supplementary material for: Bio-orthogonal click-targeting nanocomposites for chemo-photothermal synergistic therapy in breast cancer
Source: Theranostics. 2020 Apr 6;10(12):5305–21. doi: 10.7150/thno.42445 (PMC7196291; doi:10.7150/thno.42445)
Supplement: Supplementary file 1 — Supplementary methods and figures. [file thnov10p5305s1.pdf]

Supplementary Information

Bio-orthogonal click-targeting nanocomposites for chemo-photothermal synergistic therapy in breast cancer

Jianan Qiao‡, Fengchun Tian‡, Yudi Deng, Yunkai Shang, Shijie Chen, Enhao Chang, Jing Yao*

State Key Laboratory of Natural Medicines and Jiangsu Key Laboratory of Drug Stability of Biopharmaceuticals, Department of Pharmaceutics, China Pharmaceutical University, 24 Tongjiaxiang, Nanjing 210009, China.

*Author for correspondence: Tel.: +86-25-86185328; yaojing@cpu.edu.cn.

‡Authors contributed equally.

1. Synthesis of dibenzocyclooctyne-NH_2 (DBCO-NH_2).

The synthetic route of DBCO-NH_2 was shown in Figure S1.

1.1 Synthesis of 5H-dibenzo[a,d]cyclohepten-5-one oxime (1)

5-Dibenzosuberenone (48.5 mmol) was dissolved in pyridine, hydroxylamine hydrochloride (65.75 mmol) was added under refluxing and stirring in a 120 ^{o}C oil bath for 24 h. After concentration, the product was added ethyl acetate to dissolve and made to acidic by adding an appropriate amount of 10% aqueous citric acid solution. The solution was extrated by ethyl acetate. Brine was then used to wash the organic solution, followed by drying over anhydrous Na_2SO_4. After concentration and purification by column chromatography, the product (1) was obtained with a yield of 97%. ^{1}H-NMR (300 MHz, $CDCl_3$): δ 7.69-7.63 (m, 1H), 7.61-7.55 (m, 1H), 7.46-7.33 (m, 6H), 6.96-6.85 (m, 2H).

1.2 Synthesis of dibenzo[b,f]azocin-6(5H)-one (2)

Polyphosphoric acid (270 mmol) and the product (1) (13.5 mmol) were stirred at 125 ^{o}C for 30 min. Thereafter, a large amount of ice water was added to the mixture to quench the reaction, and the solution was stirred at 25 ^{o}C for 30 min. After filtration, it

was washed with deionized water. After evaporation in vacuo, the product (2) was obtained with a yield of 98%.

1.3 Synthesis of 5,6-dihydrodibenzo[b,f]azocine (3)

$LiAlH_4$ (92 mmol) was dissolved in anhydrous diethyl ether, and the product (2) (46 mmol) was added in an ice-bath, and then stirred at 40 ^{o}C for 15 h, followed by pouring into the ice water. The solution was extrated by ethyl acetate. Brine was then used to wash the organic solution, followed by drying over anhydrous Na_2SO_4. After concentration and purification by column chromatography, the product (3) was obtained with a yield of 70%. ^1H-NMR (300 MHz, $CDCl_3$): δ 7.28-7.12 (m, 4H), 7.00-6.83 (m, 2H), 6.66-6.45 (m, 3H), 6.36 (d, J=13.1 Hz, 1H), 4.57 (s, 2H).

1.4 Synthesis of 3-(2,2,2-trifluoroacetamido) propionic acid (4)

3-aminopropionic acid (30.0 mmol) was dissolved in methanol, and then triethylamine (45.0 mmol) and trifluoroacetate (45.0 mmol) were added and stirred for 15 h at 25 ^{o}C. After concentration, the product was dissolved in ethyl acetate, and then the solution was extrated by KH_2PO_4. Brine was then used to wash the organic solution, followed by drying over anhydrous Na_2SO_4. After concentration, the product (4) was obtained with a yield of 49%.

1.5 Synthesis of 3-(2,2,2-trifluoroacetamido) propionyl chloride (5)

The product (4) (10.9 mmol) was dissolved in dichloromethane, and thionyl chloride (174.1 mmol) was added and stirred at 75 ^{o}C for 15 h with nitrogen protection. After concentration, the product (5) was obtained with a yield of 86%.

1.6 Synthesis of N-(3-(dibenzo[b,f]azocin-5(6H)-yl)-3-oxopropyl)trifluoroacetamide (6)

The product (3) (19.3 mmol) was dissolved in dichloromethane, and pyridine (57.9 mmol) was added and stirred at 25 ^{o}C for 30 min. Afterward, added the product (5) under the ice bath and stirred at 25 ^{o}C for 1 h. Distilled water and brine were then used

to wash the organic solution, followed by drying over anhydrous Na_2SO_4. After concentration and purification by column chromatography, the product (6) was obtained with a yield of 91%. ^{1}H-NMR (300 MHz, $CDCl_3$): δ 7.44 (br, 1H), 7.34-7.23 (m, 4H), 7.23-7.11 (m, 4H), 6.79 (d, *J*=13.0 Hz, 1H), 6.57 (d, *J*=13.0 Hz, 1H), 5.53(d, *J*=15.3 Hz, 1H), 4.28 (d, *J*=15.2 Hz, 1H), 3.56-3.40(m, 2H), 2.33-1.97 (m, 2H).

1.7 Synthesis of N-(3-trifluoroacetamidopropanoyl)-5,6,11,12-tetrahydro-11,12-dibromodibenzo[b,f]azocine (7)

The product (6) (4.8 mmol) was dissolved in dichloromethane, and added pyridinium tribromide under the ice bath and stirred at 25 °C for 12 h with nitrogen protection. 10% citric acid aqueous solution and brine were then used to wash the organic solution, followed by drying over anhydrous Na_2SO_4. After concentration and purification by column chromatography, the product (7) was obtained with a yield of 80%. ^{1}H-NMR (300 MHz, $CDCl_3$): δ 7.73 (d, *J*=7.7 Hz, 1H), 7.58 (br, 1H), 7.25-6.87 (m, 6H), 5.91-5.77 (m, 2H), 5.16 (d, *J*=9.9 Hz, 1H), 4.20 (d, *J*=14.8 Hz, 1H), 3.76-3.49 (m, 2H), 2.73-2.31 (m, 2H).

1.8 Synthesis of N-(3-trifluoroacetamidopropanoyl)-5,6-dihydro-11,12-didehydrodibenzo[b,f] azocine (8)

Potassium tert-butoxide (5.6 mmol) was dissolved in tetrahydrofuran, and the product (7) (1.86 mmol) was added dropwise in an ice-bath and stirred for 3 h at 50 °C with nitrogen protection. 10% citric acid aqueous solution and brine were then used to wash the organic solution, followed by drying over anhydrous Na_2SO_4. After concentration and purification by column chromatography, the product (8) was obtained with a yield of 64%. ^{1}H-NMR (300 MHz, $CDCl_3$): δ 7.67 (d, *J*=7.3 Hz, 1H), 7.44-7.28 (m, 5H), 7.19-7.07 (m, 2H), 5.15 (d, *J*=14.0 Hz, 1H), 3.73 (d, *J*=14.0 Hz, 1H), 3.54-3.43 (m, 1H), 3.35-3.21 (m, 1H), 2.60-2.43 (m, 1H), 2.08-1.88 (m, 1H).

1.9 Synthesis of DBCO-NH_2

Product (8) (0.47 mmol) was dissolved in methanol, and K_2CO_3 (45.0 mmol) aqueous solution was added and stirred at 25 °C for 12 h. After concentrated, the product was dissolved in ethyl acetate. Then, distilled water and brine were used to wash the organic solution, followed by drying over anhydrous Na_2SO_4. After concentration and purification by column chromatography, the DBCO-NH_2 was obtained with a yield of 51%. ^{1}H-NMR (300 MHz, $CDCl_3$): δ 7.69 (d, *J*=7.4, 1H), 7.42-7.24 (m, 5H), 7.18-7.04 (m, 2H), 5.16 (d, *J*=13.8 Hz, 1H), 3.68 (d, *J*=13.8 Hz, 1H), 2.81-2.67 (m, 2H), 2.49-2.37 (m, 1H), 2.02-1.93 (m, 1H). HRMS, ESI$^+$, m/z: Calcd for $C_{18}H_{16}N_2O$ $(M+H)^+$, 277.13; found, 277.1335.

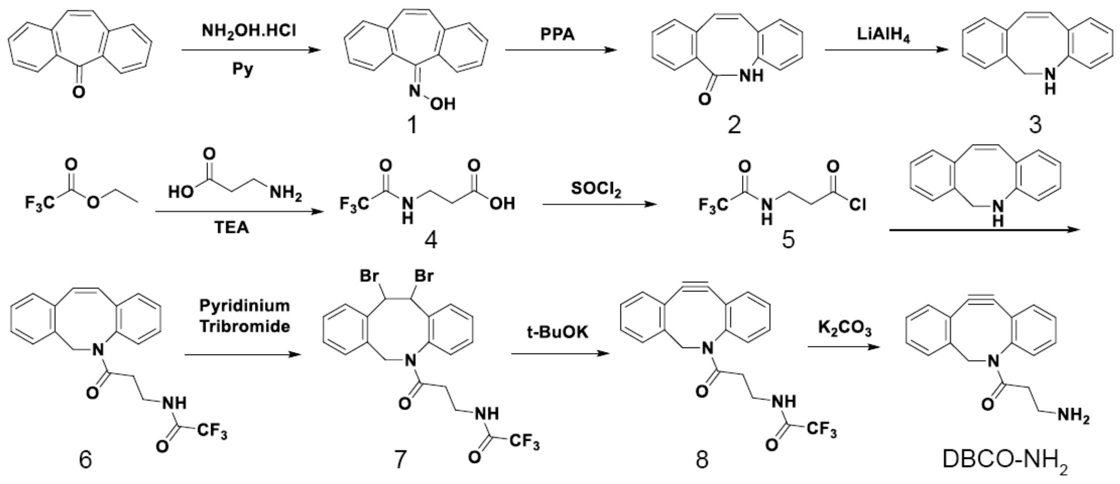

Figure S1. Synthetic scheme of DBCO-NH_2

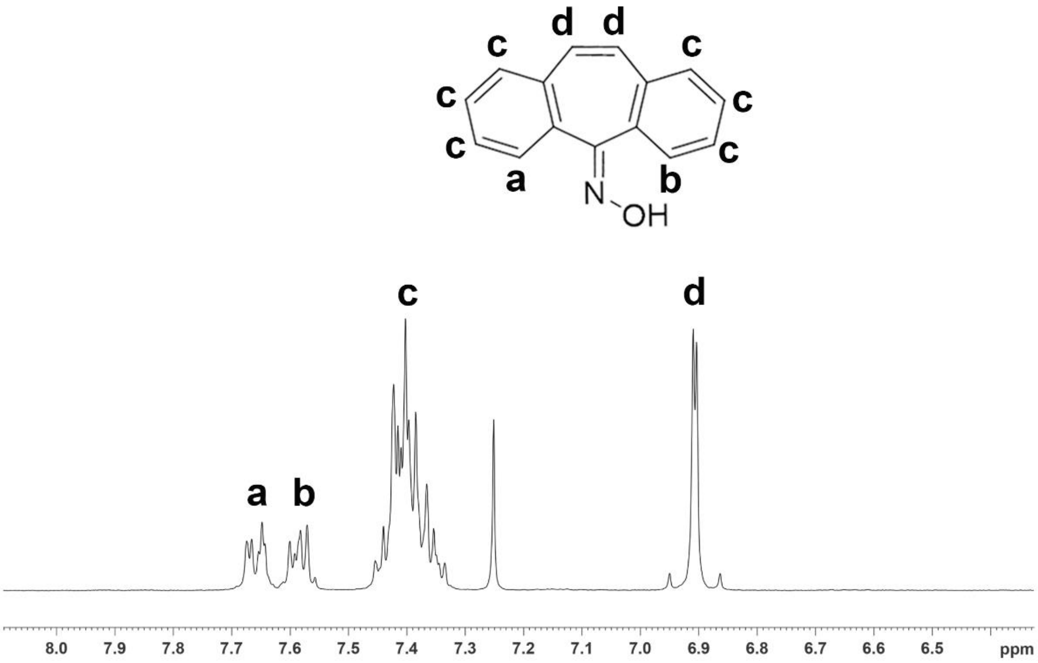

Figure S2. ^{1}H-NMR spectrum of product 1.

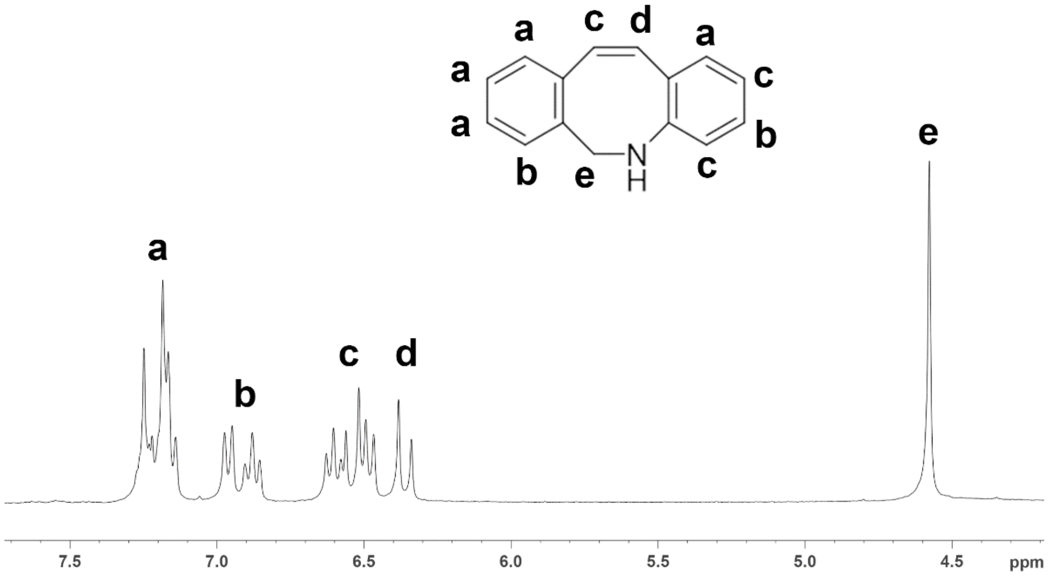

Figure S3. ^{1}H-NMR spectrum of product 3.

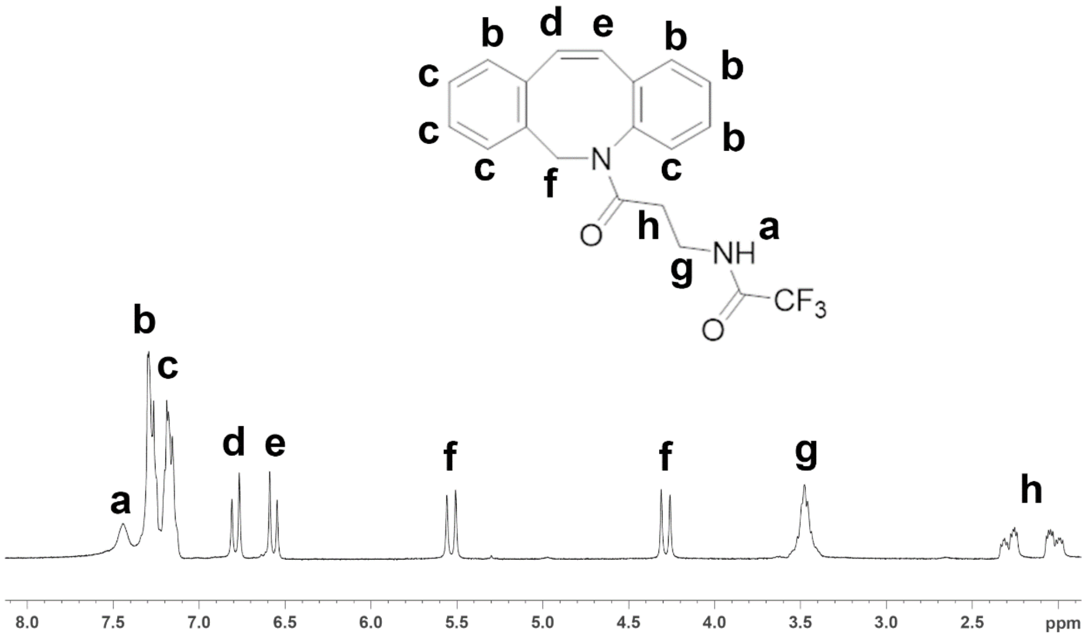

Figure S4. ^{1}H-NMR spectrum of product 6.

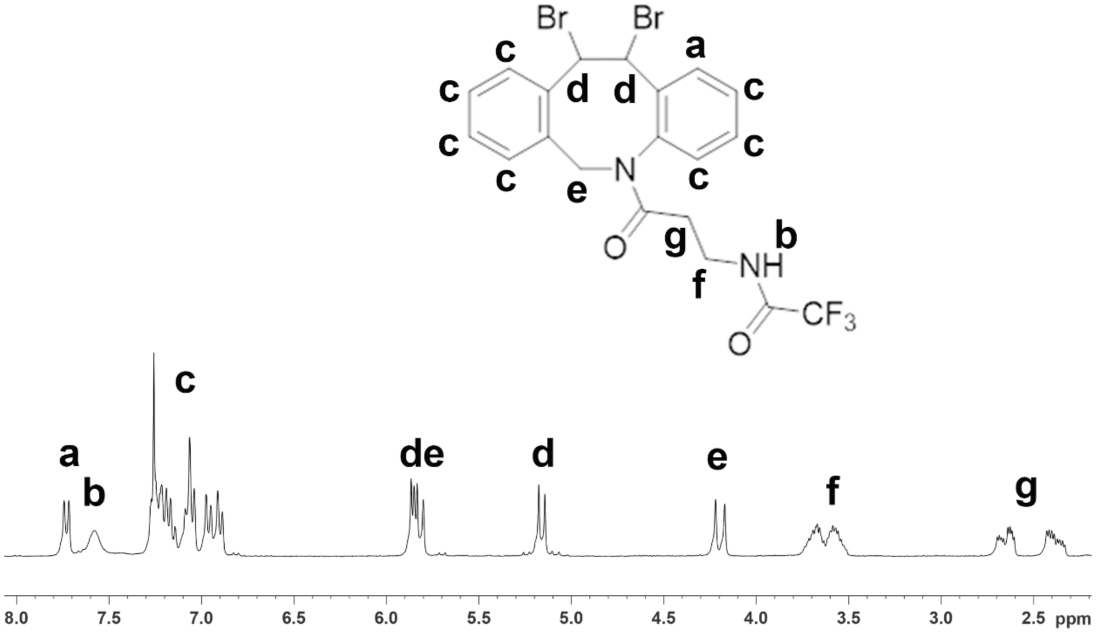

Figure S5. ^{1}H-NMR spectrum of product 7.

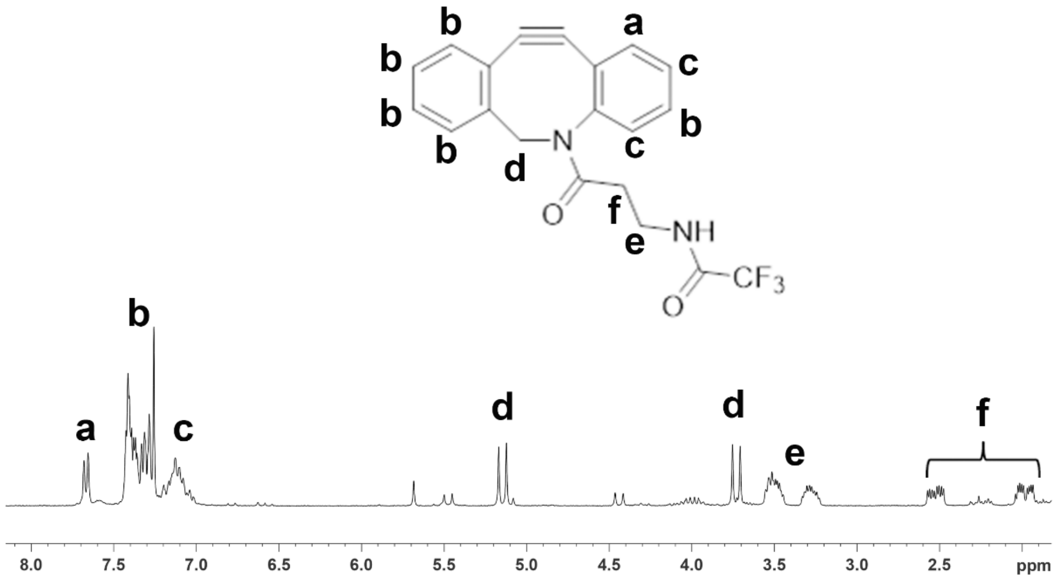

Figure S6. ¹H-NMR spectrum of product 8.

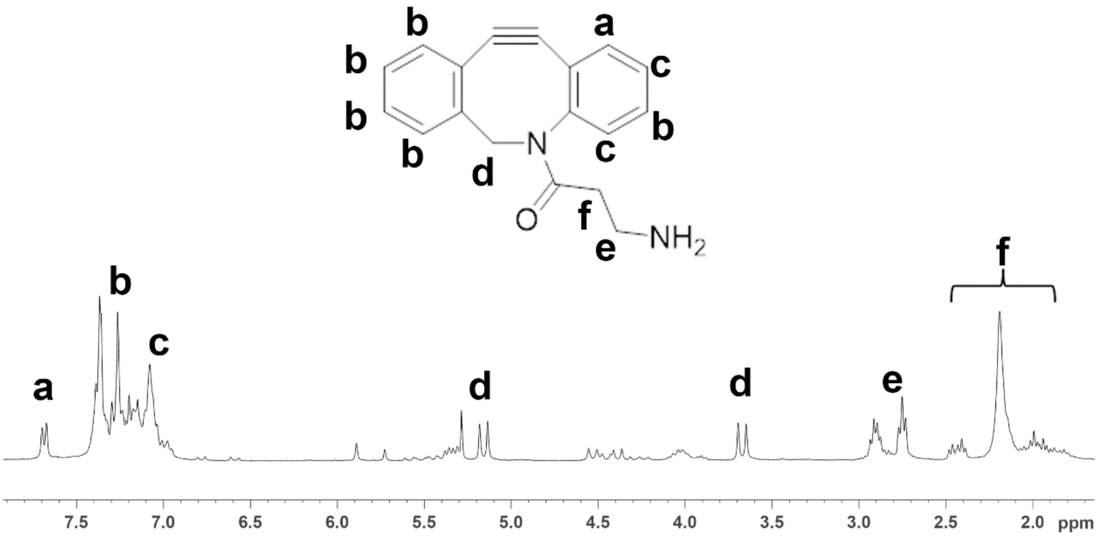

Figure S7. ¹H-NMR spectrum of product DBCO-NH_2.

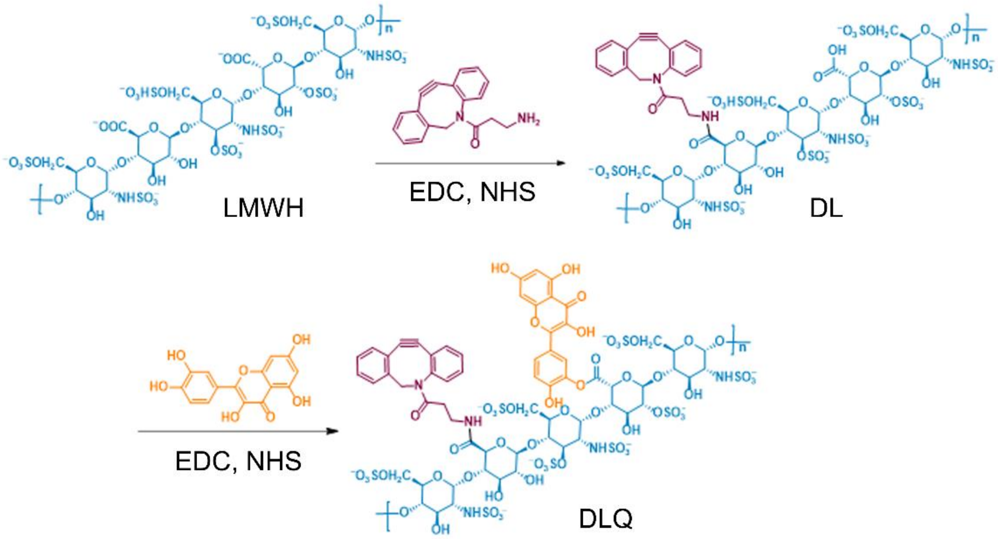

Figure S8. Synthetic scheme of DLQ conjugate. Abbreviations: EDC (1-Ethyl-3 (3-dimethylaminopropyl) carbodiimide), NHS (N-hydroxysuccinimide), LMWH (low molecular weight heparin), DL (DBCO-low molecular weight heparin conjugate), DLQ (DBCO-low molecular weight heparin-quercetin conjugate).

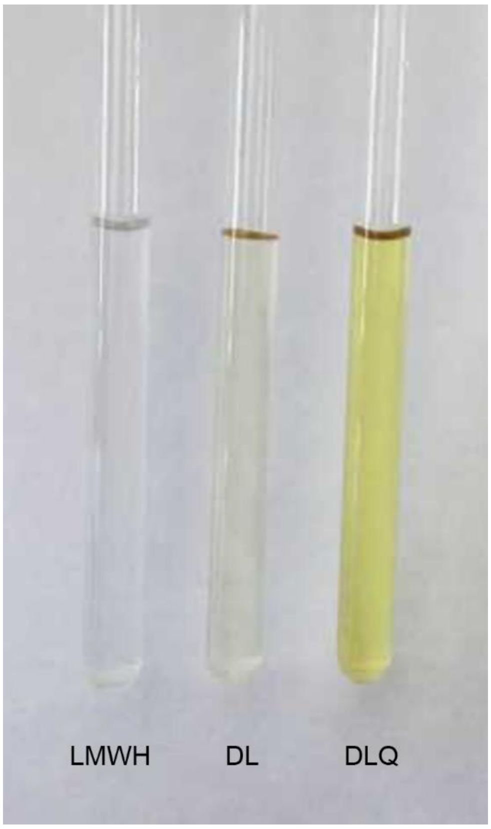

Figure S9. Representative photographs of LMWH, DL and DLQ in DMSO/water (9/1, v/v).

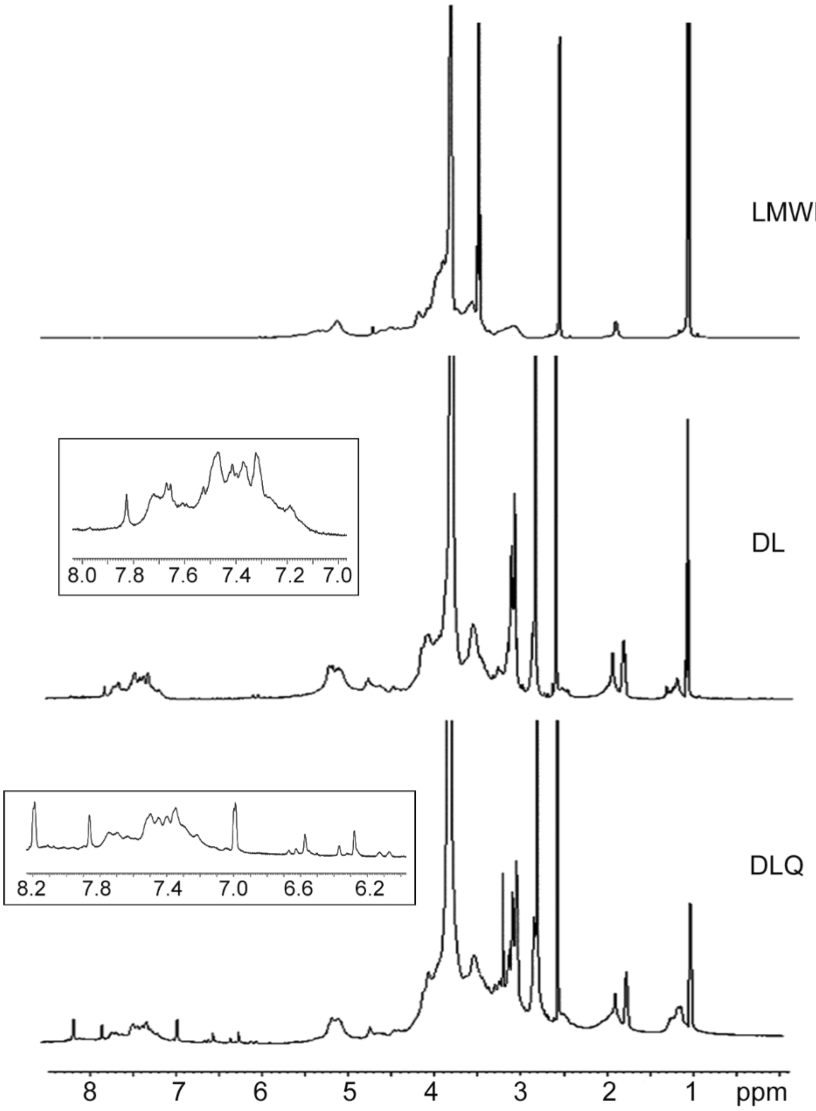

Figure S10. ¹H-NMR spectrum of LMWH, DL and DLQ in DMSO/water (9/1, v/v).

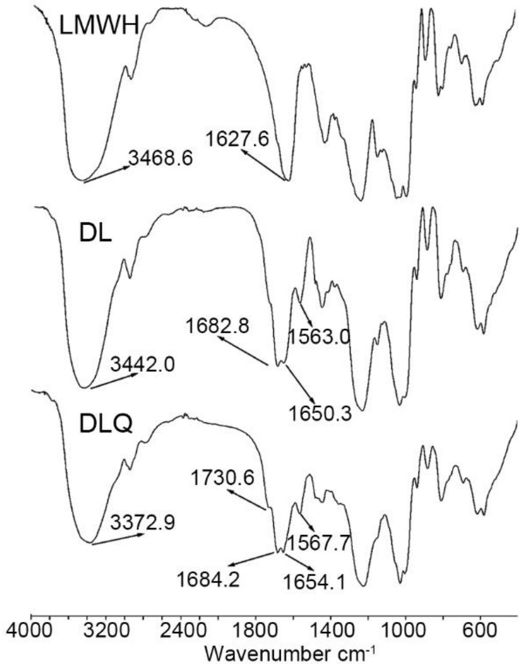

Figure S11. FT-IR spectrum of LMWH, DL and DLQ.

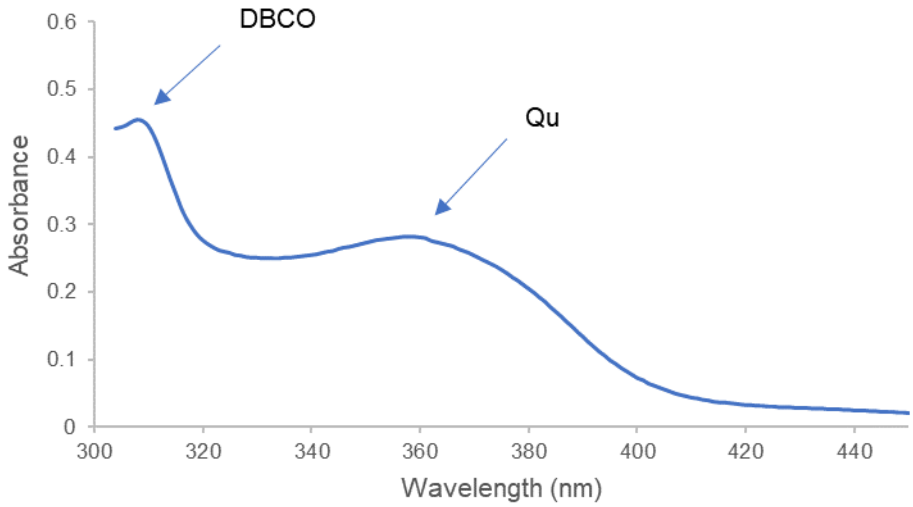

Figure S12. UV–Vis spectra of DLQ in methanol/water (9/1, v/v).

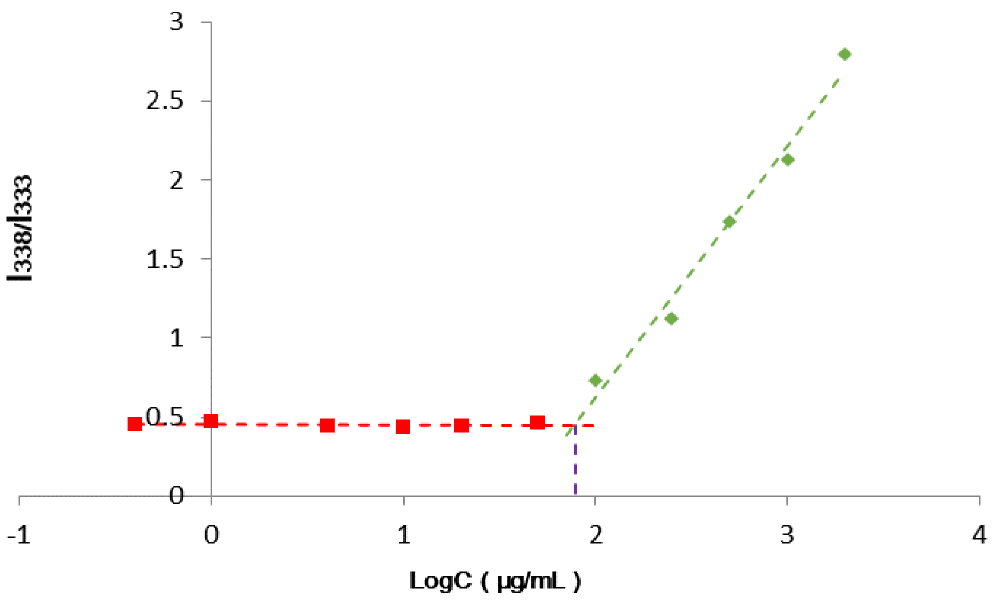

Figure S13. The CAC value of DLQ nanocomposites.

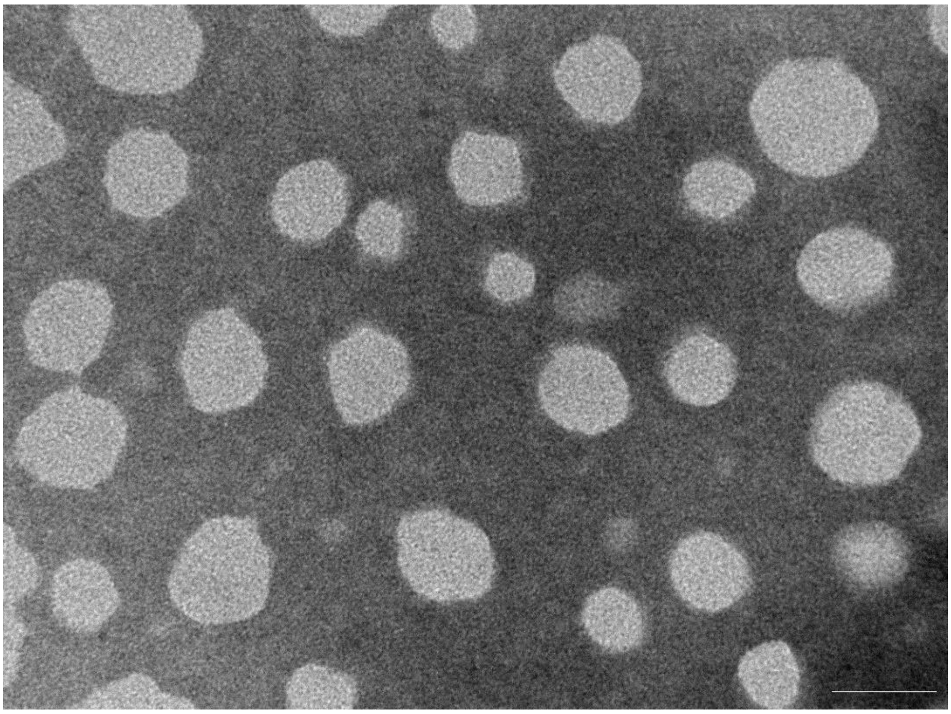

Figure S14. The transmission electron microscopy (TEM) image of DLQ/DZ nanocomposites. Scale bar is 50 μm.

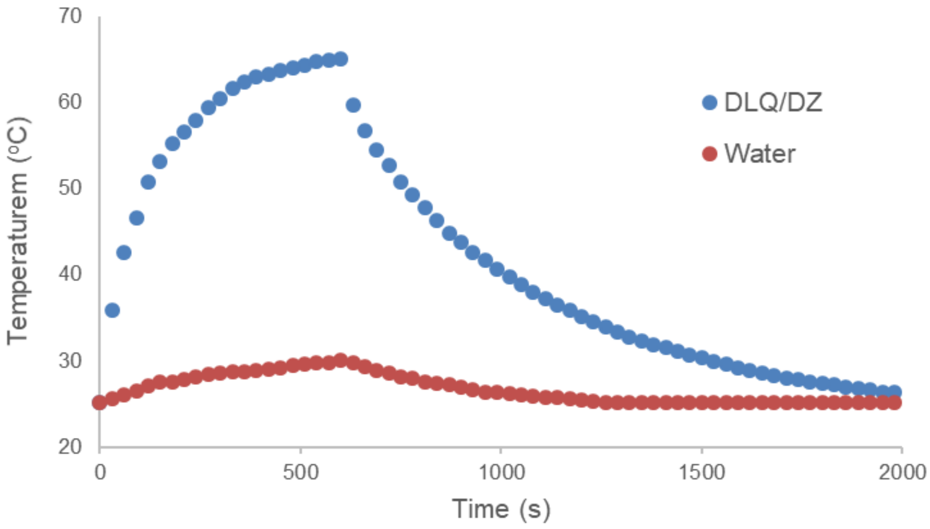

Figure S15. Photothermal heating and cooling curves of the DLQ/DZ (200 μg/mL) and pure DI water upon NIR irradiation (2 W/cm^2).

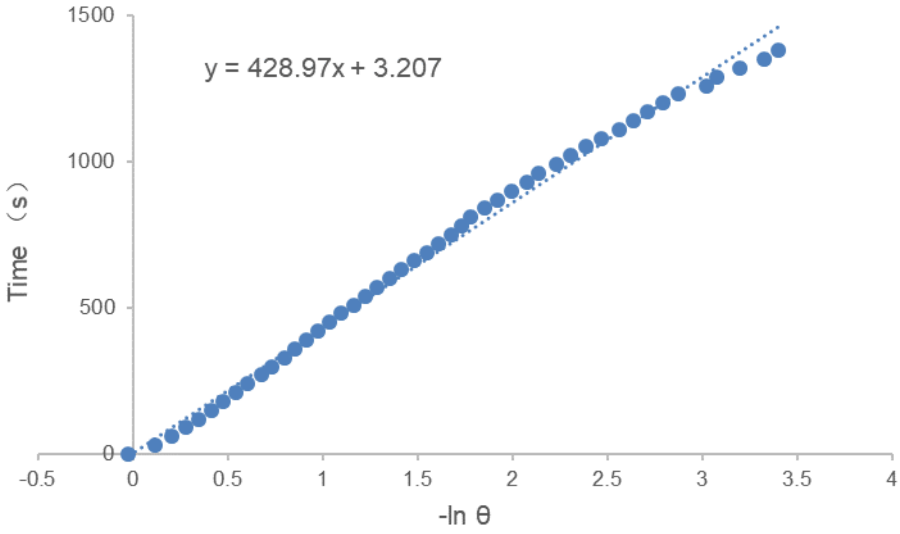

Figure S16. Linear fitting between the cooling time data versus -ln θ (the negative natural logarithm of the temperature driving force), which was calculated from the system cooling period in Figure S15.

The photothermal conversion efficiency was calculated as described in previous reports [1, 2]. Based on the results in Figure S15-16, the photothermal conversion efficiency of DLQ/DZ under 808 nm continuous laser was 21.9%. The photothermal stability of DLQ/DZ was evaluated by 808 nm laser irradiated for 10 min and turning off the laser for four cycles (Figure S17).

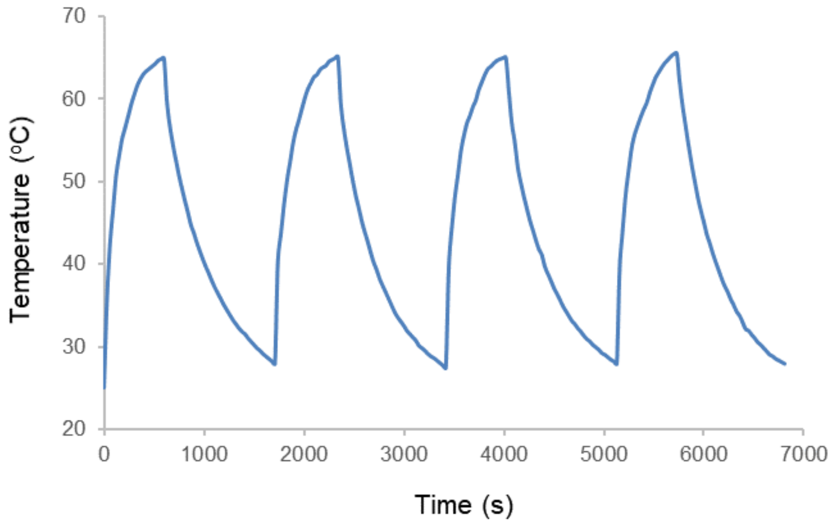

Figure S17. Temperature change of DLQ/DZ under four irradiation/cooling cycles (2 W/cm^2)

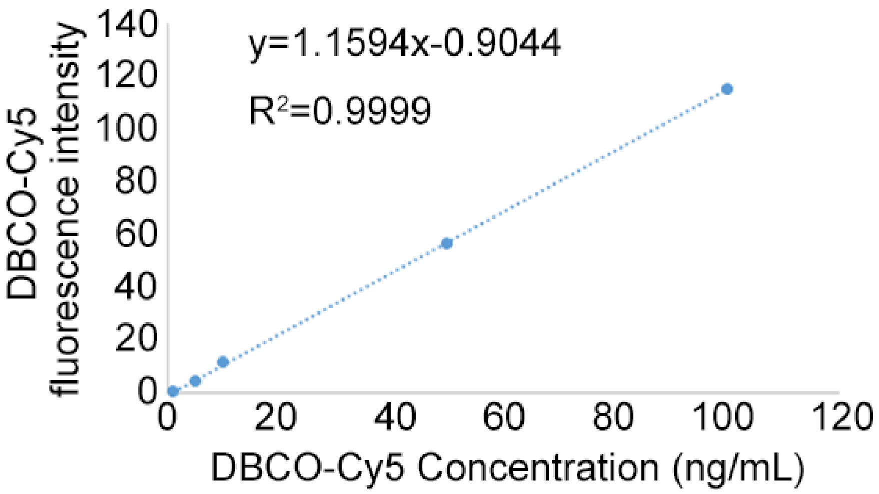

Figure S18. The standard curve of DBCO-Cy5.

Measured Cy5 fluorescence intensity of MCF-7 cells after incubated with 200 μM Ac4ManNAz for 72 h, and labeled with 50 μM DBCO-Cy5 for 1 h. According to the DBCO-Cy5 standard curve, the mass of DBCO-Cy5 can be calculated as 5.42×10^{-5} ng/cell, the moles of N_3 was 5.37×10^{-17}/cell, and the number of N_3 was 3.23×10^{7}/cell.

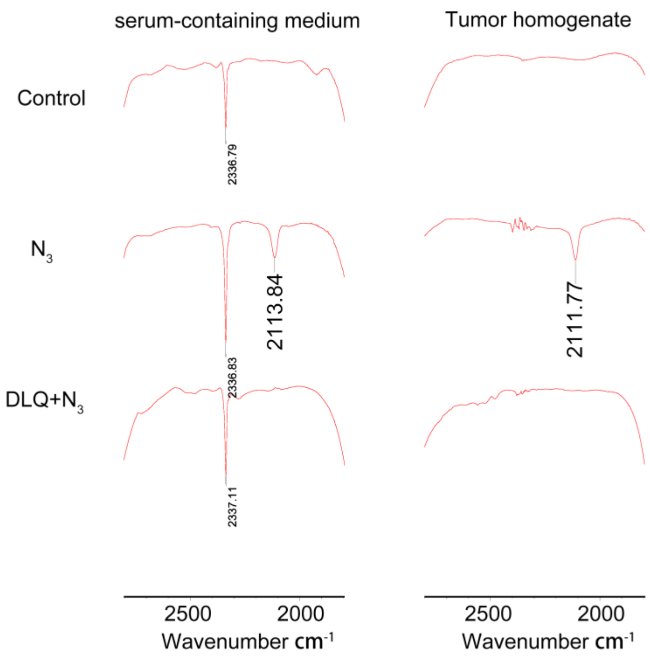

Figure S19. FT-IR spectra of N_3 and DLQ+N_3 in serum-containing medium or tumor homogenate.

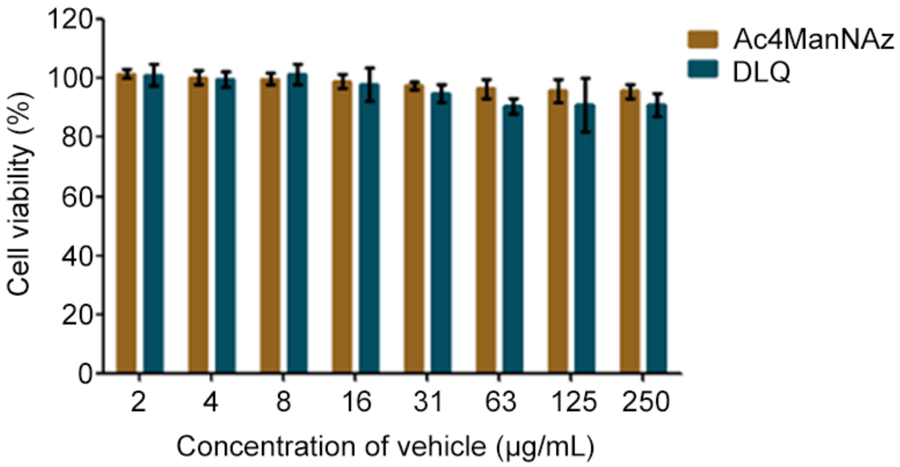

Figure S20. *In vitro* cytotoxicity of Ac4ManNAz and DLQ after treatment for 24 h. Data are represented as mean±SD (n=6).

References:

1. Liu Y, Ai K, Liu J, Deng M, He Y, Lu L. Dopamine-melanin colloidal nanospheres: An efficient near-infrared photothermal therapeutic agent for in vivo cancer therapy. Adv Mater. 2013; 25: 1353-9.
2. Zhou J, Lu Z, Zhu X, Wang X, Liao Y, Ma Z, et al. NIR photothermal therapy using polyaniline nanoparticles. Biomaterials. 2013; 34: 9584-92.
